# Measuring the dynamics of attentional capture in hand and eye movements with virtual reality

**DOI:** 10.3758/s13428-026-03177-9

**Published:** 2026-09-24

**Authors:** Max D. Gurr, Jeff Moher, Matthew D. Egbert, Christopher D. Erb

**Affiliations:** 1 School of Psychology, University of Auckland, 23 Symonds Street, Building 302, Auckland, 1010 New Zealand; 2 Department of Psychology, Connecticut College, 270 Mohegan Avenue, New London, CT 06320 USA; 3 School of Computer Science, University of Auckland, 38 Princes Street, Building 303, Auckland, 1010 New Zealand

**Keywords:** Additional singleton task, Attentional capture, Hand tracking, Eye tracking, Virtual reality

## Abstract

Laboratory investigations of attentional capture typically use two-dimensional displays and button-press responses, which may limit their generalizability to everyday experiences. The current study used a virtual reality (VR) system to measure the dynamics of attentional capture by simultaneously recording participants’ (*N* = 54) hand and eye movements as they completed a modified additional singleton task. In both a sparsely detailed and richly detailed virtual environment, participants reached to select a uniquely shaped target that was accompanied by a uniquely colored distractor on 50% of trials. We observed robust attentional capture costs across our measures of hand and eye movements, with greater latencies in initiation and movement times, larger reach curvatures, more overall eye movement, and delayed first target fixations on distractor-present trials. Three-dimensional target attraction and distractor attraction scores provided sensitive measures of the time course of attentional capture and allowed for more direct interpretations of performance than reach curvature. Although initiation times, response times, gaze distances, and target fixation proportions were elevated in the richly detailed environment relative to the sparsely detailed environment, we failed to observe a significant interaction between environmental detail and attentional capture costs in any of our measures. Taken together, our results demonstrate the feasibility of using VR to examine the dynamics of attentional capture in immersive virtual environments with richer, more continuous behavioral measures.

**Supplementary Information:**

The online version contains supplementary material available at https://doi.org/10.3758/s13428-026-03177-9.

## Introduction

Visual attention is commonly investigated in the laboratory with button-press tasks that require participants to identify a target within an array of simple shapes on a two-dimensional display (Folk et al., 1992; Gaspelin & Luck, 2018; Luck et al., 2021; Theeuwes, 1992, 2024; Wang et al., 2019). In daily life, however, we use visual attention to guide complex, prolonged interactions with three-dimensional objects, often in cluttered visual environments. Researchers have attempted to reduce the disconnect between our real-world experiences and laboratory paradigms by, for example, using eye-tracking to study how participants complete search tasks with real objects (Hout et al., 2022), developing realistic driving simulators (Chapman & Underwood, 1998; Garrison & Williams, 2013), and translating tasks featuring two-dimensional images into immersive three-dimensional virtual environments (Beitner et al., 2021, 2024; Harada et al., 2025; Olk et al., 2018).

Researchers have also sought to reduce the disconnect between the real world and the laboratory by replacing traditional button-press measures of performance with three-dimensional hand-tracking methods that enable participants to respond by reaching to target locations (e.g., Erb et al., 2022; Moher et al., 2015; Welsh, 2011; Welsh & Pratt, 2008; Wood et al., 2011). Hand-tracking methods, however, have their own limitations. For instance, the electromagnetic position and orientation recording systems commonly used to record three-dimensional reaching movements in visual attention tasks are costly and sensitive to ferromagnetic metals, making the systems difficult to use with standard digital displays and eye-tracking systems and limiting their portability for research outside of the laboratory. Additionally, much of the work that has used three-dimensional hand-tracking methods to investigate visual attention has continued to rely on tasks featuring simple stimuli presented on two-dimensional displays.

To address these limitations, the current study examined the extent to which a commercially available virtual reality (VR) headset could be used to investigate the dynamics of attentional capture in hand and eye movements. In addition to supporting simultaneous hand and eye tracking in immersive virtual environments, VR headsets are relatively affordable and portable when compared to traditional tracking technologies. In the following, we briefly review the literature on button-press measures of attentional capture in the additional singleton task (Theeuwes, 1991, 1992), highlighting research investigating how the distance between targets and distractors modulates capture costs. We then review previous hand- and eye-tracking work with the paradigm before introducing our VR version of an additional singleton task.

### Attentional capture

Attentional capture occurs when salient information is automatically attended to, disrupting one’s ability to attend and respond to task-relevant information. Attentional capture is often studied using an additional singleton task (Theeuwes, 1991, 1992) in which participants search for a unique target (e.g., a shape singleton such as a diamond amongst circles) that is occasionally presented in the presence of a salient distractor (e.g., a color singleton such as a green shape amongst red shapes). In the mixed feature version of the task (Theeuwes, 1991), the defining feature of the unique target is not known ahead of time (e.g., the unique shape could be a diamond or a circle on any given trial). In the fixed feature version (Theeuwes, 1992), the defining feature of the unique target is prespecified (e.g., the unique shape is a diamond on each trial). Performance on distractor-present trials is generally impaired, exhibiting higher error rates and slower response times (RTs), relative to distractor-absent trials (Folk, 2013; Geyer et al., 2008; Pinto et al., 2005; Theeuwes, 1991, 1992; Theeuwes et al., 2008).

Most studies investigating attentional capture in the additional singleton task have measured the impact of distractors on performance in terms of button-press measures of error rate and RT (Folk, 2013; Gaspelin et al., 2015, 2017; Geyer et al., 2008; Lien et al., 2022; Pinto et al., 2005; Theeuwes, 1992; Theeuwes et al., 2008; Wang & Theeuwes, 2020; Wang et al., 2019). This work indicates that performance is impaired when task demands are high, such as when the shape and color of the distractor is randomized so that the target-defining feature is not known in advance (Gaspelin et al., 2015, 2017; Leonard & Egeth, 2008; Leonard & Luck, 2011; Pinto et al., 2005; Theeuwes et al., 2003), or when extra objects are added to the search display (Wang & Theeuwes, 2020). These task demands require a “singleton detection mode” (Bacon & Egeth, 1994), in which attention focuses on unique features. Accordingly, using singleton detection mode activates both a unique target shape and a unique color distractor.

In contrast, distractor effects can be greatly reduced or eliminated entirely when the shape and color of the distractor are kept constant (Gaspelin et al., 2015; Leonard & Luck, 2011; Lien et al., 2022; Moher & Leber, 2025; Müller et al., 2009; Wang & Theeuwes, 2020), when the distractor is presented more often at a specific location (Wang & Theeuwes, 2020; Wang et al., 2019), or when the heterogeneity of the distractors prevent singleton detection mode and force participants to use a “feature search mode” (Bacon & Egeth, 1994) where attention can be oriented towards an expected feature, such as a shape or color, without activating unexpected distractors. Accordingly, when using feature search mode, or a fixed feature version of the additional singleton paradigm that allows for the potential suppression of salient distractors, susceptibility to attentional capture by salient distractors appears to decrease.

Using a circular array of shapes, Hickey and Theeuwes (2011) and Gaspar and McDonald (2014) tested the effects of target-distractor proximity. To isolate the immediate attentional effects of salient distractors, participants in both experiments were instructed to maintain central fixation throughout each trial. For both defined target features (Gaspar & McDonald, 2014) and randomized target features (Hickey & Theeuwes, 2011), distracting stimuli caused the greatest RT increase when they appeared directly adjacent to the target singleton. Hickey and Theeuwes (2011) found that the performance costs observed in RTs decreased as the distance between the target and the distractor increased. Additionally, when controlling for the distance separating the target and distractor, the researchers found that the effect of the distractor was significantly smaller when the target and distractor were presented in separate visual hemifields as opposed to the same hemifield. Similarly, Gaspar and McDonald (2014) observed a significant decrease in attentional capture costs in RTs as target-distractor distance increased, with no evidence of attentional capture when the target and distractor were presented on opposite sides of the circular array. These results indicate that attentional capture by a distracting stimulus is strongly influenced by its spatial proximity to the target, with larger distances or placement in opposite hemifields reducing capture costs or precluding capture altogether.

### Hand and eye tracking

To explore how the temporal and spatial dynamics of attentional capture unfold over time, researchers are increasingly using continuous measures of hand and eye movements (e.g., Erb et al., 2022; Gaspelin et al., 2017, 2019; Irwin et al., 2000; Moher et al., 2015; Theeuwes et al., 1998, 2003; Wang et al., 2019; Welsh, 2011; Zhang et al., 2025). For example, Welsh (2011) measured participants’ hand movements as they completed an attentional capture task by reaching to touch targets on a digital display. Welsh observed that participants’ reach movements were curved toward the location of an attention-capturing but invalid cue, indicating that attentional capture triggered response activations aligned with the cue’s position. Importantly, this effect only emerged when the cue’s salient dimension (abrupt onset or color) matched that of the target, mirroring earlier findings in button-press measures (e.g., Folk et al., 1992). Welsh (2011) interpreted the observed impact of attentional capture on reaching movements to be consistent with action-centered models of attention (e.g., Rizzolatti et al., 1994; Tipper et al., 1992) proposing that action and attention are reciprocally linked such that to-be-performed actions influence the distribution of attention and, conversely, the distribution of attention influences the production of actions (Welsh & Zbinden, 2009). Subsequent studies exploring the additional singleton paradigm with reach movements have similarly demonstrated that salient distractors that do not share the salient dimension of the target can disrupt not only the timing of action responses but also the movement trajectories themselves (Kerzel & Schönhammer, 2013; Moher et al., 2015).

Erb et al. (2022) used three-dimensional reach tracking to investigate the development of attentional capture across childhood, adolescence, and early adulthood with an additional singleton task that featured the simultaneous presentation of a shape singleton target and a color singleton distractor. Movement times (MTs) revealed significant attentional capture costs in children but not in adolescents or young adults, suggesting that older participants may have been able to avoid attentional capture. However, reach trajectories showed significant attentional capture effects in each age group tested, with capture effects not differing across age groups. Thus, spatial characteristics of performance showed comparable capture costs across development, while temporal characteristics showed significant age-related reductions in capture costs, suggesting that older participants offset capture effects by increasing their speed during corrective movements.

Building on earlier work from Van Zoest and Donk (2008), Gaspelin et al. (2017) investigated how the presence of distractors impacts eye movements by measuring the first saccade made by participants when locating a shape singleton in the presence of a color singleton distractor. When the target and distractor features were randomized between trials, thereby encouraging singleton detection mode (Bacon & Egeth, 1994), first saccades were equally likely to be directed towards the shape singleton target and the color singleton distractor. However, when the target shape was kept the same on each trial (e.g., diamond) and multiple non-target shapes were presented (e.g., squares and hexagons), thereby encouraging feature search mode, first saccades were more likely to be directed towards the target singleton and away from the color distractor. Wang et al. (2019) further showed that biasing the distractor to a specific location reduced initial eye movements toward the high-frequency distractor location, regardless of whether a distractor or target was present there.

Consistent with manual measures of performance, eye tracking measures indicate that the adoption of feature search mode may allow participants to preferentially ignore distractors in favor of the target shape when initiating visual search (Gaspelin et al., 2017; Geyer et al., 2008; Moher et al., 2011; Wang & Theeuwes, 2020; Wang et al., 2019). However, when forced to use singleton detection mode, attention is biased towards salient features, drawing eye movements towards distractors (Gaspelin et al., 2017, 2019) and slowing responses (Gaspar & McDonald, 2014; Gaspelin et al., 2017; Hickey & Theeuwes, 2011; Wang et al., 2019).

Although studies investigating the dynamics of attentional capture with hand movements or eye movements have provided important insights into visual attention, this work has still largely relied on tasks featuring simple, two-dimensional stimuli presented in a narrow visual range against a uniform background. Additionally, many of the studies that have investigated visual attention by recording three-dimensional hand movements have subsequently analyzed movement trajectories in two dimensions (e.g., Erb et al., 2022; Moher et al., 2015; Welsh, 2011), often focusing on summary measures reflecting the extent to which the observed trajectory deviated from a direct path to the selected target (e.g., max deviation or movement curvature). Finally, few studies investigating attentional capture have collected continuous measures of hand and eye movements simultaneously (e.g., Kerzel & Schönhammer, 2013; Song & McPeek, 2009), possibly due to the technological challenges associated with combining certain techniques (e.g., screen-mounted eye-tracking systems are typically mounted to the bottom of the screen, meaning that reach movements to the screen will obstruct the eye-tracking cameras) or the costs associated with purchasing hand- and eye-tracking systems.^1^ Ideally, researchers investigating the dynamics of attention and distraction would be able to measure hand and eye movements simultaneously in tasks presented in rich, three-dimensional environments.

### Current study

Commercially available VR systems provide the ability to record hand and eye movements simultaneously as participants complete tasks in three-dimensional environments. In addition to being relatively affordable and portable, VR systems can be used to present tasks in highly controlled immersive virtual environments designed to mimic the complex, dynamic environments we experience in daily life (Maymon et al., 2023; Parsons, 2015). Consequently, VR is increasingly being used within the psychological and brain sciences to investigate topics including spatial navigation (Starrett et al., 2021; Warren et al., 2017), the impact of fear on the experience of presence (Maymon et al., 2024), and the joint Simon effect (Harada et al., 2025). Head-mounted VR displays have also been used to investigate visual search by, for example, studying how participants use a controller-contingent window to search a virtual room for a target object, akin to using a flashlight to search a dark room (Beitner et al., 2024). Recent work has also used VR to measure hand and eye movements as participants completed a three-dimensional Color Trails Test (CTT), a trail-making test commonly used to assess sustained visual attention (Wilf et al., 2024). However, little research has used VR to investigate the dynamics of attention and distraction in hand and eye movements.

The current study was therefore designed to evaluate the suitability of using a commercially available VR system to measure hand and eye movements as participants completed a modified version of the additional singleton paradigm in a sparse virtual environment designed to mimic a laboratory environment and in a rich virtual environment portraying a greenhouse. Specifically, we addressed the following three research questions:

1. *How do the manual and oculomotor dynamics of attentional capture function in three-dimensional environments?* If (a) the hand and eye tracking measures afforded by current VR systems provide suitable measures of performance and (b) the attentional capture effects observed in two-dimensional contexts generalize to three-dimensional environments, we should observe robust attentional capture effects when comparing distractor-present and distractor-absent trials.
2. *Can we improve upon maximum deviation measures derived from two-dimensional trajectories by considering how three-dimensional movements unfold over time?* Although hand-tracking measures based upon maximum deviation or area under the curve calculations allow for efficient summaries of the extent to which a hand movement deviated from a direct trajectory to the selected target, these measures do not capture how competition among response alternatives unfolds over time. We therefore developed three-dimensional target attraction and distractor attraction measures to capture what proportion of a reach movement is directed towards a given stimulus.
3. *To what extent is performance on the additional singleton task impacted by the richness of the virtual environment?* Existing research with two-dimensional displays indicates that visual search performance is impaired by cluttered visual environments (Semizer & Michel, 2019; Semizer & Rosenholtz, 2025). This work suggests that performance should generally decrease (e.g., RTs should increase) as the richness of the three-dimensional visual environment increases. However, it is possible that three-dimensional shape and depth cues may allow participants to separate objects from a cluttered background more readily in VR relative to a two-dimensional display. It is also unclear whether the richness of the environment impacts attentional capture by, for example, increasing or decreasing the difficulty of disengaging attention from a salient distractor.

## Method

### Participants

Sample sizes for experiments investigating attentional capture typically range from 20 to 30 participants. However, given the lack of previous research using VR to investigate attentional capture with hand and eye movements, we aimed to collect a minimum of 50 participants to ensure adequate power to detect a medium effect. The final sample included 54 participants between 18 and 26 years of age (*M* = 18.8 years, *SD* = 1.4 years, 14 males, 40 females, 0 gender diverse). All participants included in the final sample had corrected-to-normal vision, understood English, were capable of normal reaching behavior, had no diagnosed cognitive or social impairments, and were willing to wear a VR headset. Seven additional participants were excluded for equipment failure (*n* = 1), not meeting the inclusion criteria (*n* = 2), and generating an extreme number of errors (*n* = 2) or extremely slow responses (*n* = 2). Three participants included in the final sample had missing or incomplete eye-tracking data and, consequently, were excluded from all gaze analyses.

Participants were culturally diverse, with 16 participants (29.6%) identifying as New Zealand European, 16 participants (29.6%) identifying as Asian, seven (12.9%) participants identifying as Other European, three (5.6%) participants identifying as Middle Eastern, Latin American, or African, three (5.6%) participants identifying as Pacific Peoples, three (5.6%) participants identifying as Other (not specified), two (3.7%) participants identified as New Zealand European and Pacific Peoples, two (3.7%) participants identified as New Zealand European and Māori, one (1.9%) participant identified as both Māori and Pacific Peoples, and one (1.9%) participant identified as New Zealand European and Middle Eastern, Latin American, or African. Participants were recruited through the University of Auckland School of Psychology research pool and received course credit for participation. All participants signed a written consent form and completed a demographic survey before the experiment. The University of Auckland Human Participants Ethics Committee approved the experiment (UAHPEC 22063).

### Materials

The experiment was conducted using a head-mounted VR headset (HMD, HTC Vive Pro Eye with 2 × dual-OLED 3.5" screens, resolution 1440 × 1600 per eye, 90 Hz, 110° FOV) in combination with two HTC Vive Base Stations (version 2.0) to track participants' head and hand movements. An HTC VIVE Pro Hand-Held Controller was held in each participants’ dominant hand and was used to interact with objects in the virtual environment. Reach movements and response selections were measured once per display frame within the Unity application at a rate of approximately 90 Hz. The eye-tracking in the headset operates at 120 Hz with 0.5–1.1° accuracy but was recorded once per display frame within the Unity application using the HTC SRanipal runtime and Tobii XR Software Development Kits (SDK), at a rate of approximately 90 Hz.

The experiment was developed and run using Unity (2021.3.45f1) on a Dell PC with an Intel(R) Core(TM) i7-9700 CPU, NVIDIA GeForce RTX 2080 GPU, 32 GB RAM, and Windows 10 Pro operating system. The virtual environment and experimental stimuli were created using assets from the Unity Asset Store and Blender for additional modeling. The Unity Experiment Framework (2.0; Brookes et al., 2020), a package for developing behavioral experiments in Unity, was used to organize the experimental structure and data collection. The task and materials used in the experiment are available at https://github.com/CMND-Lab/VR-SingletonCapture_ViveProEye_OSF.

**Fig. 1:**
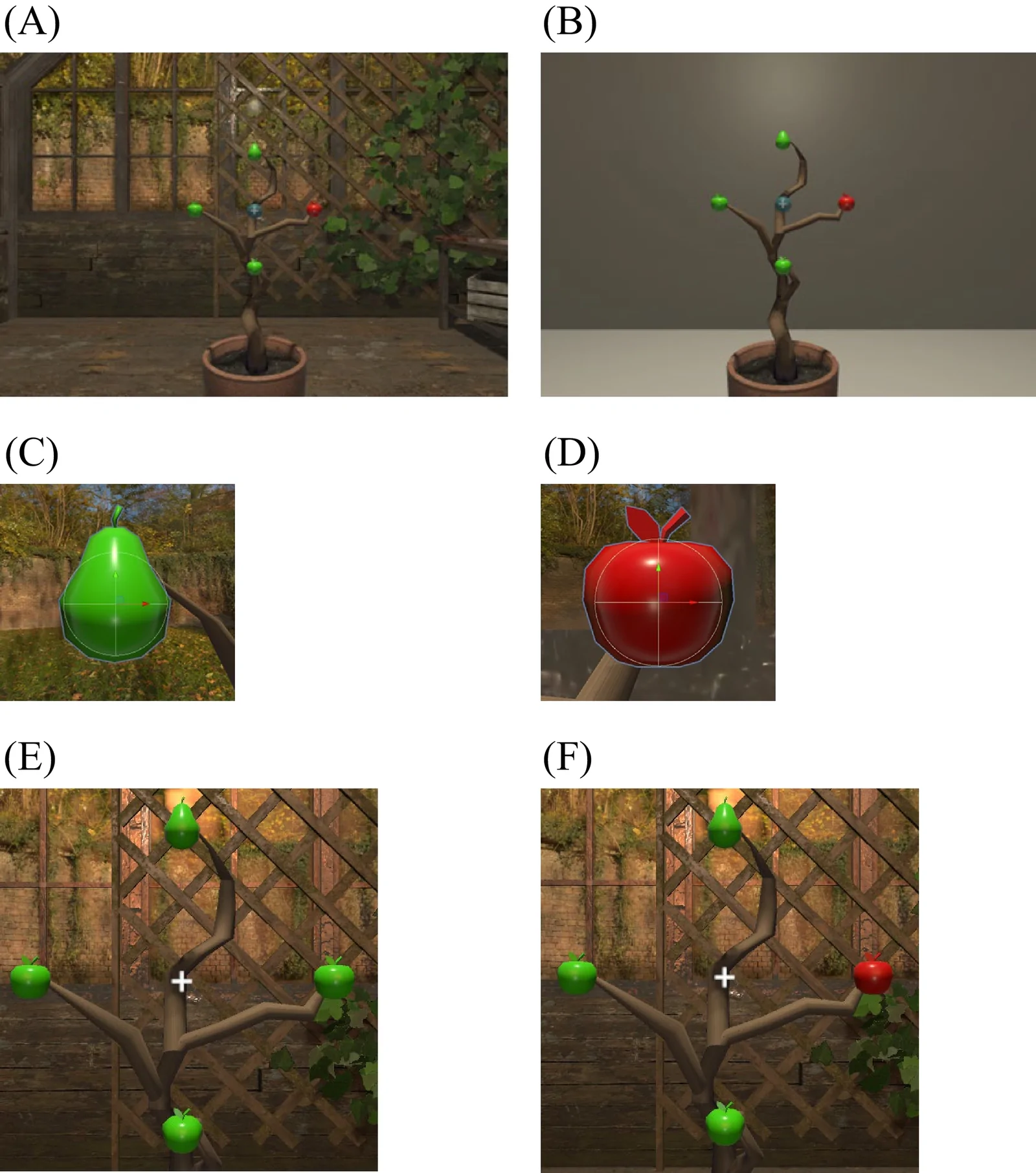
Experimental stimuli and environment setup. *Note.* **(A)** Sparsely detailed room condition during a standard trial. **(B)** Richly detailed room condition during a standard trial. **(C)** and **(D)** A green pear-shaped stimulus and a red apple-shaped stimulus, both with an interactive response sphere in the center (5 cm in diameter). **(E)** Illustration of a distractor-absent trial with three apple-shaped non-targets and a green pear-shaped target. **(F)** Illustration of a distractor-present trial with three apple-shaped non-targets, a green pear-shaped target, and a red color distractor

Each participant completed the experiment in two virtual environments. The sparsely detailed control environment consisted of a medium-sized, well-lit, untextured, 4.25-m × 4.5-m room without a ceiling (see Fig. 1A). The color of the walls was HSV(0, 0, 32.2), and the floor color was HSV(0, 0, 91.4). The walls were 2.5 m tall, and participants were positioned in the center of the room. The richly detailed environment (see Fig. 1B) consisted of a well-lit, 8.8-m × 7.7-m greenhouse made of assets from the Unity Asset Store (Unity Technologies, 2026). Participants were positioned in this environment at 2 m from the left wall, and 2 m from the front wall. In the real world, participants were seated in the center of the experimental room, facing a wall located approximately 1.5 m from the participant's chair. The environment was changed for each participant halfway through the experiment, with the presentation order counterbalanced across participants.

Within the virtual environment, participants completed a modified version of the mixed-feature singleton detection task (Theeuwes, 1991) in which a uniquely shaped target (approximately 5 cm in diameter) and three non-target stimuli (approximately 5 cm in diameter) were presented at cardinal locations (top, left, right, bottom) directly in front of the participant (see Fig. 1C, D). Each set of stimuli used 3D models of apples and pears, taken from the Unity Asset Store, with the target object appearing in a unique shape (e.g., a pear target presented with three apples, or an apple target with three pears). The stimuli were placed on a model of a potted tree (see Fig. 1A, B), created in Blender. We adjusted the tree and stimulus presentation height to each participant’s eye level at the beginning of the experiment.

On each trial, the target (uniquely shaped) object was presented at one of three locations (left, top, right) on a branching tree, with a non-target object shown at each of the remaining cardinal locations (top, left, right, bottom). For example, if the target object were an apple presented at the top location, non-target pear objects would be presented at the left, right, and bottom locations. In distractor-absent trials (see Fig. 1E), all shapes were presented in the same color, either green HSV(101, 100, 100) or red HSV(0, 100, 100). In distractor-present trials (see Fig. 1F), one of the non-target shapes presented in the left, right, or top locations appeared in the opposite color to the target object. The target was never presented in a unique color. To encourage the adoption of singleton detection mode (Bacon & Egeth, 1994), the target shape and color were randomized on each trial. Given that the bottom response location was partially obscured by the participant’s virtual hand and controller, neither the shape target nor color distractor was presented at this location. Although the position of the virtual objects could have been altered to allow the starting orb to appear in the middle of the target locations, doing so would have obstructed the presentation of the fixation cross and made it more difficult to judge the relative depth of the starting orb and target locations. Given that previous hand-tracking research observed significant capture costs when the presentation of the target and distractor were restricted to the left, top, and right locations (e.g., Erb et al., 2022), we opted to allow the bottom response location to be partially occluded by the starting orb.

In each block of 48 trials, participants encountered 24 distractor-absent trials and 24 distractor-present trials in a randomized order. There were 12 unique distractor-absent trial types, reflecting the three target locations (left, top, or right), the two target shapes (pear or apple), and the two target colors (red or green). Each of these distractor-absent trial types was presented twice within a block. There were 24 unique distractor-present trial types, reflecting the six target-distractor configurations (e.g., target at the left, distractor at the top), the two target shapes (pear or apple), and the two target colors (red or green). Each of these distractor-present trial types was presented once within a block.

### Procedure

Before the experiment began, a stylized blackboard with instructions was presented 1.9 m in front of participants. During this instruction sequence, participants completed a five-point eye-tracking calibration procedure to ensure the headset was positioned correctly and to adjust the height of the experimental display. This calibration procedure was repeated if participants removed the headset during any of the breaks between blocks. Participants were then given a baseline block of 12 trials requiring them to reach for a target appearing alone at the left, right, or top location. Participants then completed a practice block of 12 experimental trials, using the format described above. Before the onset of the experiment, participants were reminded to respond only to the unique shape and were encouraged to act quickly with the instruction, “Please respond as quickly as you can while avoiding making errors.”

Participants interacted with the experiment using a white HSV(0, 0, 100) sphere (2.5 cm in diameter) positioned on top of the virtual controller. Each trial was initiated by participants holding the controller sphere inside a centrally located sphere (4.5 cm in diameter, 8.5 cm below eye level, and 15 cm in front of the participant) for a total of 2 s. After the first second of holding the controller inside the starting location, a white fixation cross (5 cm in width and height, 8.5 cm below eye level, and 45 cm in front of the participant) appeared in the center of the stimuli layout (see Fig. 1E, F). At the end of the 2-s holding period, the fixation cross disappeared, and four stimuli appeared 22 cm from the top, left, right, and bottom of the fixation cross position. If participants moved the controller outside of the central starting sphere before the stimuli appeared, the 2-s holding period would stop and restart when the participant returned the controller to the central sphere. Following the appearance of the stimuli, the participant had 3 s to make a response in the experimental trials, with no time limit given during the practice block. Responses were made by moving the controller sphere from the starting location to one of the four stimuli, with each trial ending 50 ms after the controller sphere intersected with any of the stimuli, or after the 3-s time limit. If the correct response was made (i.e., the controller intersected with the uniquely shaped object), a high tone of 600 Hz was played through speakers located next to the participant. If an incorrect response was made, or if the participant failed to respond in time, a low tone of 300 Hz was played.

The experiment consisted of 6 experimental blocks, with each block consisting of 48 trials. Half of the participants completed the practice block and first three experimental blocks in the greenhouse (richly detailed) environment, whereas the other half of the participants completed the practice block and first three experimental blocks in the control (sparsely detailed) environment. The instructions and baseline block always occur in the greenhouse environment. After three experimental blocks, participants removed the headset and took a short break (5 min). Following the break, participants completed the eye calibration procedure and completed the final three experimental blocks in the alternate environment.

### Data processing

Hand-tracking data were pre-processed following the methods outlined by Moher and Song (2013) and implemented using custom Python and MATLAB software. A data file that specified the size and spatial orientation of the object locations for the experiment (the four stimuli, the starting orb, and the fixation cross) was loaded into custom Python software, and the eye level of each participant was input to adjust the height of each object. The hand-tracking data were then resampled to a uniform rate of 90 Hz, following the methods outlined by Moher and Song (2013). This resampling process involved removing endpoint effects by detrending the original signal, resampling the data to 90 Hz, and adding the trend back in. The data were then filtered with a low-pass Butterworth filter (dual pass, 10-Hz cutoff, 2nd-order).

Each participant's hand-tracking data were individually screened using an automatic algorithm to detect movement onset and movement completion. Following Valevicius et al. (2018), movement onset was identified using a combination of a speed threshold and a space threshold. The speed threshold identified the first point on each trial at which hand movement speed exceeded 5% of the maximum velocity produced during the reach movement within the trial. The space threshold identified the first point during the trial that the controller left the starting orb. Initiation time (IT) on each trial was calculated using the speed threshold unless the speed threshold identified a later point in time than the space threshold (e.g., if the participant happened to begin their reach movement especially slowly and left the orb before increasing their speed), in which case the space threshold was used. Movement completion times for each trial were recorded during the experiment when a response was detected (i.e., the time at which the controller intersected one of the stimulus objects). For each trial, the point in the resampled reach trajectory nearest to the recorded completion time was designated as the endpoint of that trajectory.

Following the automatic detection of movement onset and completion, the movement trajectories and velocity profiles for each trial were visually inspected for errors and inaccuracies, and the appropriate thresholds were either adjusted (e.g., if the algorithm identified a small swaying movement soon after the start of the trial as the point of movement onset, the movement onset threshold was manually adjusted based upon plotted velocity thresholds and three-dimensional trajectories) or the trial was marked for exclusion from analysis (e.g., if the plotted movement trajectories indicated sampling issues on the trial resulting in gaps in the movement). On average, 11.85% (*SD* = 8.07%) of valid experimental trials (trials with a recorded response) were manually adjusted, and 1.19% (*SD* = 1.76%) of valid experimental trials were manually excluded for each participant. Manual processing was completed while blind to the experimental conditions on each trial, and the frequency of manual adjustments per participant did not differ significantly between distractor-absent trials (*M* = 11.6%, *SD* = 8.4%) and distractor-present trials (*M* = 12.1%, *SD* = 8.0%), *t*(53) = – 1.276, *p* = .21, or between the sparse room (*M* = 11.8%, *SD* = 8.1%) and the rich room (*M* = 11.9%, *SD* = 9.5%), *t*(53) = 0.073, *p* = .94.

**Fig. 2:**
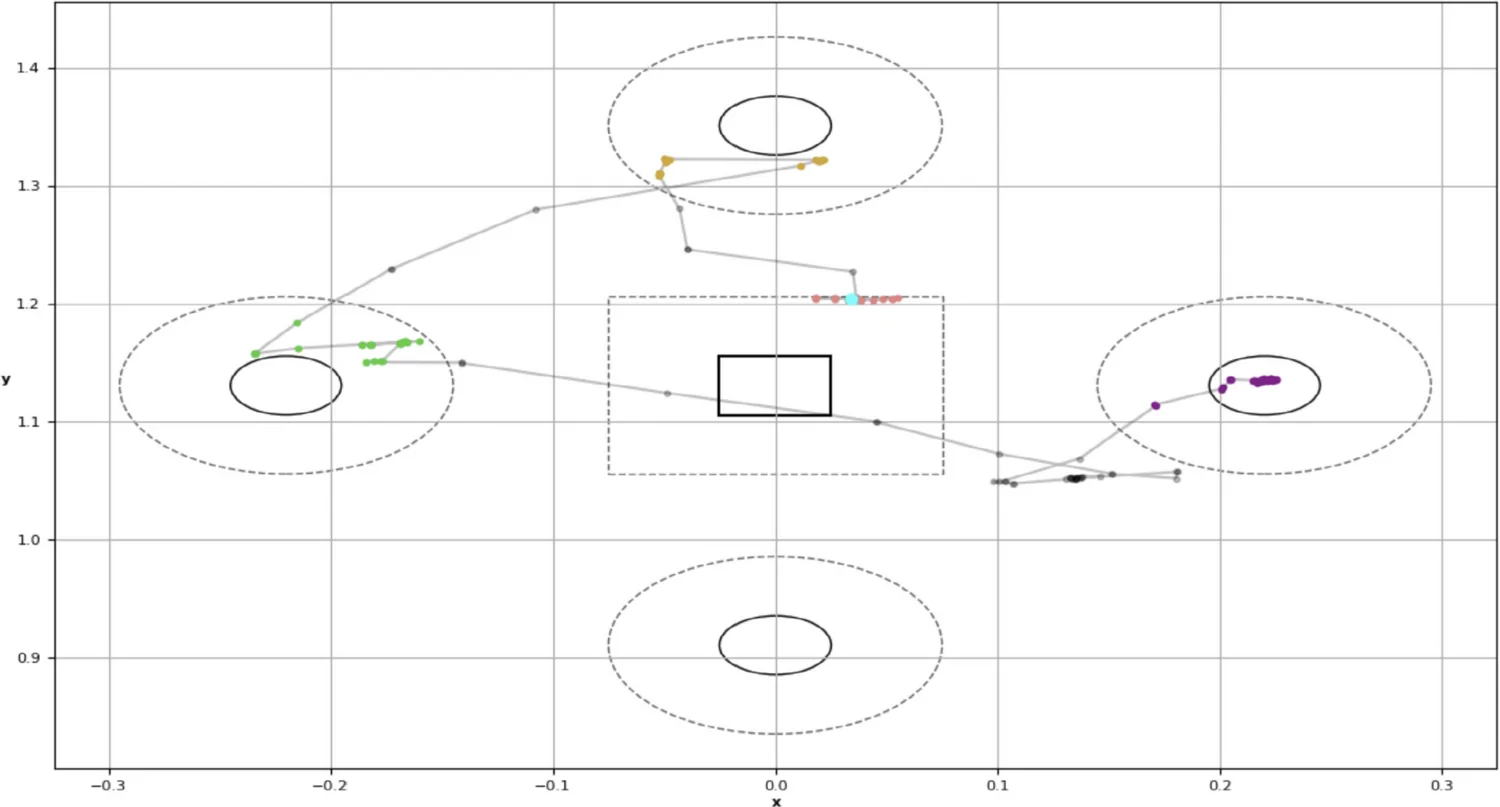
Data Processing ROIs with fixation points. *Note*: Example 2D representation of the gaze intersections recorded for a trial with the target at the right location and a distractor at the top location, with the *x* and *y* axes presenting horizontal and vertical space (in meters), respectively. *Shapes with solid black outlines* represent the actual sizes of objects in the experiment. *Shapes with dashed black outlines* represent the tolerance regions used for focus detection

Eye-tracking data were recorded as a single 3D gaze vector representing gaze origin and direction. Within the custom Python software, regions of interest (ROIs) were created by adding a 5 cm tolerance around each of the specified object locations. For each timepoint of each trial, intersections were calculated in 3D space between the gaze vector and each of the ROIs, excluding the starting orb (see Fig. 2 for a 2D representation). If an intersection was detected with an ROI, that timepoint was labeled as a fixation on the relevant object. To account for jitter in recording, small gaps (< = 100 ms) between fixations on the same ROI were filled in. Similarly, to account for eye movements passing over objects, small periods of fixation on an ROI (< = 50 ms) were removed.

### Dependent variables

After movement onset and movement completion thresholds were finalized, measures of IT, MT, and RT were calculated, with IT calculated as the time elapsed between stimulus onset and movement onset, MT calculated as the time elapsed between movement onset and movement completion, and RT calculated as the time elapsed between stimulus onset and movement completion. Movement trajectories were then normalized using a method based on functional data analysis (FDA) described by Gallivan and Chapman (2014). Broadly, the FDA-based method to normalize across reach distance consists of three steps: (1) a trajectory is fit with a mathematical function using B-splines and is smoothed using a roughness penalty, (2) the trajectory is then oversampled in time to extract the position and times that correspond to equally spaced points along the reach distance, and (3) the three-dimensional data corresponding to those distance-normalized time values are extracted to generate the final space-normalized trajectory (for more details, see Gallivan & Chapman, 2014).

When using FDA, data is to be normalized to a dimension that does not significantly vary across experimental conditions. As the regions in space where participants started and ended their hand movements (starting marker and targets) are always the same along the *z*-axis of Unity’s reference frame, we selected the z dimension. Following the conventional number of time steps in the normalization procedure in mouse-tracking (Freeman & Ambady, 2010) and reach-tracking studies (Moher & Song, 2014), we normalized the trajectories to have 101 data points.

Trajectories for calculating curvature (CURV) were measured in two-dimensional *xy* space by calculating maximum perpendicular deviation (MPD). This index is commonly calculated as the length of the perpendicular line from a straight-line trajectory to the farthest sample on the reach movement trajectory from such an idealized direct path (Hehman et al., 2015). The straight-line trajectory is drawn from the sample corresponding to movement onset to the sample corresponding to movement termination. Following Erb et al. (2022), the measure is dimensionally construed by scaling the largest perpendicular distance by the length of the idealized trajectory and taking the absolute value of the result.

In addition to calculating CURV, the three-dimensional spatial characteristics of reaching behaviors were analyzed by calculating “target attraction” and “distractor attraction” measures. These measures were calculated by computing the distance to each response object for each timepoint. Between each consecutive pair of timepoints, the object with the largest decrease in distance was labeled as the object “attracting” the hand at that timepoint. Reach “attraction” towards each object location was then calculated for each trial by calculating the proportion of timepoints exhibiting attraction toward each of the objects. Target attraction (TA) was calculated for each trial as the reach attraction towards the target location (i.e., the proportion of timepoints in the trial where the hand was moving more towards the target object than any other object). Distractor attraction (DA) was calculated for each distractor-present trial as the reach attraction towards the distractor location (i.e., the proportion of timepoints in the trial where the hand was moving more towards the distractor object than any other object). DA differences were then calculated, for each distractor-present trial, as the difference between attraction towards the distractor location and the average attraction towards the same location on distractor-absent trials, in the same room, with the same target location.

Three primary gaze measures were calculated. Gaze distance (GD) was calculated as the summed angular distance (in radians) between each gaze vector during the trial, providing an index of the overall amount of eye movement on each trial. First target fixation (FTF) was calculated for each trial as the start of the first fixation period (> 100 ms) on the target ROI. Target fixation proportion (TFP) was calculated as the proportion of time the participant looked at the target object during the trial. This was calculated as the total amount of time during the trial where the 3D gaze vector intersected with the target ROI, divided by the trial duration.

Prior studies have used the location of first saccades (identified as an eye movement exceeding a velocity of 30–35°/s and acceleration of 9500°/s^2^) as a measure of immediate attention allocation towards a target or distractor location (e.g., Geyer et al., 2008; Irwin et al., 2000; Moher et al., 2011; Theeuwes et al., 1998, 2003). We originally planned to use this measure, but it became apparent during data processing that participants tended to adopt a consistent search strategy, with more than 50% of first saccades going to the top location for 72.5% of participants, the right location for 7.8% of participants, the left location for 9.8% of participants, and the bottom location for 9.8% of participants.^2^ First saccades did not seem to be affected by distractor presence, as the strategy used by each participant remained consistent on both distractor-absent trials (*M* = 66.3%, *SD* = 18.6%) and distractor-present trials (*M* = 65.1%, *SD* = 19.1%).

## Results

The analyses presented in this manuscript were not pre-registered. The data and R scripts used to perform the analyses reported in this manuscript are available at https://osf.io/hcz4m.

Average error rates were low in both the rich room (*M* = 2.09%, *SD* = 1.85%) and sparse room (*M* = 1.85%, *SD* = 1.91%) and, as such, were not analyzed further. Low error rates are commonly observed in reaching tasks, as participants can detect and override incorrect responses (e.g., Erb et al., 2020). Following Erb et al. (2022), we excluded the first trial of each block, trials with inaccurate responses, and all trials preceded by an inaccurate response. Additionally, trials were excluded from all analyses if the average sampling frequency during the experiment was below 80 Hz, if the RT was greater than three standard deviations from the average RT for that participant, or if the trial was manually marked for exclusion during data processing. Overall, this resulted in an average of 7.7% (*SD* = 4.0%) of trials being cut for each participant.

### Preliminary analyses

We conducted a series of preliminary ANOVAs to evaluate whether the secondary factors of room order (sparse room first, rich room first), target shape (apple, pear), target color (red, green), and target location (top, right, left) interacted with our primary independent variables of distractor presence (present, absent) and room (rich, sparse). To control family-wise error rate, Holm–Bonferroni corrections were applied separately within each effect featuring a secondary factor and a primary independent variable (secondary factor × distractor presence, secondary factor × room, secondary factor × distractor presence × room), treating the eight dependent variables (IT, MT, RT, CURV, TA, FTF, GD, and TFP) as a family for each effect (α = .05 per family). Note that DA was excluded from these analyses because it was calculated as a difference score between distractor-present and distractor-absent trials in a manner that controlled for room and target location. Greenhouse–Geisser corrections were applied to all tests that violated the assumption of sphericity.

Follow-up *t* tests were conducted to further explore significant effects from each ANOVA. Given that the room order factor varied between participants, independent-samples *t* tests were used to examine these effects, while paired-samples *t* tests were used for the remaining within-subjects effects. To control the familywise error rate, Holm-Bonferroni corrections were applied separately to the follow-up *t* tests for each factor, treating each factor's comparisons as a distinct family (e.g., follow-up tests examining target location effects were corrected to reflect the three comparisons performed). The full results of these analyses are provided in Section 1 of the Supplementary Materials. Here, we report all significant interaction effects from these preliminary analyses that featured either room or distractor presence, with all reported *p* values featuring Holm–Bonferroni adjustments.

Preliminary analyses revealed an interaction between room type and room order in ITs, *F*(1, 52) = 11.57, *p* = .006 η_p_^2^ = .18, RTs, *F*(1, 52) = 13.65,* p* = .003, η_p_^2^ = .21, and GDs, *F*(1, 49) = 22.49, *p* < .001, η_p_^2^ = .32. Follow-up tests revealed no effect of room type when participants first completed the search task in the sparse room (*p* values >.57) and a significant effect of room type when participants first completed the search task in the rich environment (*p* values <.001), with faster ITs, faster RTs, and smaller GDs in the sparse environment relative to the rich environment (see Table 1).

**Table 1:** Effects of room order and room type

		Initiation time (IT)		Response time (RT)		Gaze distance (GD)	
		Sparse Environment	Rich Environment	Sparse Environment	Rich Environment	Sparse Environment	Rich Environment
Order	*Sparse First*	694 ± 140 ms	701 ± 152 ms	1220 ± 157 ms	1231 ± 182 ms	2.18 ± 0.43 rad	2.21 ± 0.48 rad
	*Rich First*	659 ± 128 ms	746 ± 168 ms	1150 ± 157 ms	1259 ± 197 ms	1.96 ± 0.39 rad	2.41 ± 0.45 rad

Each cell displays the mean and the standard deviation as a function of room order (sparse first vs. rich first) and room type (sparse environment vs. rich environment)

Preliminary analyses also revealed an interaction between distractor presence and target location in RTs, *F*(2, 106) = 9.90, *p* < .001, η_p_^2^ = .16, TAs, *F*(2, 106) = 5.18, *p* = .049, η_p_^2^ = .09, and GDs, *F*(2, 100) = 5.11, *p* = .049, η_p_^2^ = .09. Follow-up tests on RTs and TAs revealed significant distractor effects at each target location (*p* values < .001), and a significantly larger effect of distractor presence when the target was at the top location relative to the left or right locations (*p* values < .05), but no significant difference when comparing trials in which the target was at the left location to trials in which the target was at the right location. Follow-up tests on GDs revealed significant distractor effects for trials with the target at the left (*p* < .05) or top (*p* < .001) locations, but no effect at the right location. The effect of distractors was significantly larger when the target was at the top location relative to the right location (*p* < .05), but no significant differences were observed when comparing trials in which the target was at the left to trials in which the target was at the right or top.

### Planned analyses

Our primary analyses consisted of a series of repeated-measures ANOVAs featuring room type (rich, sparse) and distractor presence (present, absent) as within-subject factors for all measures aside from DA. DA was calculated as a difference score reflecting performance on distractor-absent and distractor-present trials and was therefore analyzed using (1) one-sample *t* tests to determine whether average DA scores differed significantly from zero and (2) paired-samples *t* tests to determine whether DA scores differed significantly between the rich and sparse virtual environments. To control family-wise error rate, Holm–Bonferroni corrections were applied separately within each effect type, treating the nine dependent variables as a family for the effects of distractor presence (or the distractor effect for DA) and the interaction of distractor presence and room type (or the comparison of distractor effects between rooms for DA), and the eight dependent variables (not including DA) as a family for the effect of room type (α = .05 per family). We only report these effects below if they remained significant after Holm–Bonferroni correction, with any effects not explicitly reported failing to reach significance (see Section 2 of the Supplementary Materials for all results, including non-significant effects). All reported *p* values are adjusted to reflect the Holm-Bonferroni corrections.

#### Distractor presence

As summarized in Table 2, all the dependent measures tested with an ANOVA aside from target fixation proportion revealed significant main effects of distractor presence, with distractor present trials showing slower ITs, *F*(1, 53) = 18.12, *p* < .001, η_p_^2^ = .26, MTs, *F*(1, 53) = 52.18, *p* < .001, η_p_^2^ = .50, and RTs, *F*(1, 53) = 87.66, *p* < .001, η_p_^2^ = .62, elevated curvatures, *F*(1, 53) = 50.24, *p* <.001, η_p_^2^ = .49, lower target attraction scores, *F*(1, 53) = 75.57, *p* < .001, η_p_^2^ = .59, slower first target fixation times, *F*(1, 50) = 27.86, *p* < .001, η_p_^2^ = .36, and larger gaze distances, *F*(1, 50) = 24.27, *p* < .001, η_p_^2^ = .33, than distractor absent trials. The distractor effect also differed significantly from zero for DA, *t*(53) = 8.94, *p* < .001, η_p_^2^ = .60.

**Table 2:** Mean values for distractor-absent and distractor-present trials

Comparison	Temporal			Reach			Gaze		
	IT	MT	RT	CURV	TA	DA	FTF	GD	TFP
Distractor	***	***	***	***	***	***	***	***	ns
*Absent*	692 ± 18 ms	499 ± 12 ms	1191 ± 21 ms	0.119 ±.005	92.4 ± 0.58%	NA	594 ± 21 ms	2.17 ± 0.06 rad	31.2 ± 0.87%
*Present*	708 ± 20 ms	530 ± 14 ms	1238 ± 24 ms	0.142 ±.006	89.7 ± 0.73%	+ 1.90 ± 0.21%	622 ± 23 ms	2.23 ± 0.06 rad	30.9 ± 0.89%

Each cell displays the mean and the standard error of the mean for each dependent variable: initiation time (IT), movement time (MT), response time (RT), reach curvature (CURV), target attraction (TA), distractor attraction (DA), first target fixation (FTF), gaze distance (GD), and target fixation proportion (TFP). No value is presented for the distractor-absent condition for DA because the measure requires a distractor to be present for the attraction score to be computed. ***p <.001

Although our preliminary analyses did not reveal a significant interaction between distractor presence and target location in FTFs (*p* = .25), the majority of participants adopted a strong bias to fixate on the top object first, as discussed in the Dependent variables section above. This bias may complicate the interpretation of first target fixations, as FTFs will be systematically faster for these participants on trials in which the target is presented at the top location. To examine this possibility further, we present exploratory analyses in a subsequent section that account for the relative location of the target and distractor when assessing distractor effects.

#### Room

As summarized in Table 3, performance in the rich room exhibited slower ITs, *F*(1, 53) = 13.34, *p* = .003, η_p_^2^ = .20, slower RTs, *F*(1, 53) = 16.43, *p* = .001, η_p_^2^ = .24, higher GDs, *F*(1, 50) = 18.75, *p* < .001, η_p_^2^ = .27, and higher TFPs, *F*(1, 50) = 16.22, *p* = .001, η_p_^2^ = .25, relative to performance in the sparse room. However, each of these main effects, aside from that of TFPs was qualified by a significant interaction with room order in our preliminary analyses, as reported above.

**Table 3:** Mean values for the rich and sparse rooms

Comparison	Temporal			Reach			Gaze		
	IT	MT	RT	CURV	TA	DA	FTF	GD	TFP
Room	**	ns	**	ns	ns	ns	ns	***	**
*Rich*	724 ± 22 ms	521 ± 14 ms	1245 ± 25 ms	0.130 ±.005	91.1 ± 0.66%	+ 1.74 ± 0.30%	602 ± 24 ms	2.31 ± 0.07 rad	32.1 ± 0.88%
*Sparse*	676 ± 18 ms	509 ± 13 ms	1185 ± 21 ms	0.131 ±.006	91.1 ± 0.72%	+ 2.06 ± 0.25%	615 ± 22 ms	2.08 ± 0.06 rad	29.9 ± 0.94%

Each cell displays the mean and the standard error of the mean for each dependent variable: initiation time (IT), movement time (MT), response time (RT), reach curvature (CURV), target attraction (TA), distractor attraction (DA), first target fixation (FTF), gaze distance (GD), and target fixation proportion (TFP). **p <.01, ***p <.001

#### Room by distractor presence interactions

None of the ANOVAs revealed significant interactions between room and distractor presence (all *p* values >.61), and the paired-samples *t* test comparing DA scores between the two rooms did not reveal a significant effect (*p* >.99). We supplemented our ANOVA analyses with Bayes factors, which index the relative likelihood of our observed data under the alternative hypothesis compared to the null hypothesis. All Bayes factors were computed using Faulkenberry’s (2022) *PsyStat* app (https://tomfaulkenberry.shinyapps.io/psystat), which uses the observed *F-*statistic to compute a Bayes factor directly from an ANOVA result (Faulkenberry, 2021; Faulkenberry & Brennan, 2023). Three measures revealed moderate evidence for the null (IT: BF_01_ = 4.19; RT: BF_01_ = 4.64; TFP: BF_01_ = 3.40), while the remaining measures revealed anecdotal evidence for the null (MT: BF_01_ = 1.29; CURV: BF_01_ = 1.77; TA: BF_01_ = 2.52; FTF: BF_01_ = 1.42; GD: BF_01_ = 1.34).

#### Interim discussion

Each of our dependent measures, aside from TFP, revealed robust effects of distractor presence, with significant impairments to performance on distractor-present relative to distractor-absent trials. These results indicate that the attentional capture effects observed in standard two-dimensional contexts generalize to three-dimensional VR environments. Although CURV, TA, and DA all revealed robust and highly significant effects of distractor presence, TA and DA provided more directly interpretable measures of performance, reflecting the extent to which participants’ movements were attracted to the target or the distractor over time. By contrast, CURV reflects one time point (the point of maximum deviation from a direct trajectory to the selected response target) and increased CURV values need not reflect attraction to the distractor object. Thus, our results suggest that our three-dimensional movement trajectory measures are sensitive to attentional capture effects and may be preferable to CURV.

Finally, our analyses failed to reveal any significant interactions between the richness of the virtual environment and the presence or absence of a distractor, with Bayes approximations revealing moderate evidence for the null in IT, RT, and TFP. These findings indicate that the differences in the richness of the virtual environments presented in the current experiment were not sufficient to significantly impact the size of attentional capture costs. However, MT, CURV, TA, FTF, and GD revealed only anecdotal evidence for the null, and so the evidence for the null effect is mixed and restricted to specific measures. As we explore in further detail in the Discussion, it is possible that such a modulation of attentional capture costs could occur under different conditions.

### Exploratory analyses

Our preliminary analyses revealed a significant interaction between target location and distractor presence in RTs, TAs, and GDs, with follow-up analyses demonstrating larger distractor effects when the target was at the top location. Given that the distractor was always near the target on these trials (i.e., located at the left or right location), this interaction could have stemmed from a target-distractor proximity effect (e.g., Gaspar & McDonald, 2014; Hickey & Theeuwes, 2011). To examine this possibility, we conducted exploratory analyses that separated trials into four distractor types:

- None: No distractor present in the display
- Near.Side: The distractor is at the side (left/right) and the target is at the top
- Near.Top: The distractor is at the top, and the target is at the side (left/right)
- Far: The distractor is at one side of the display (left/right), and the target is at the opposite side

To control for the main effects of target location reported above, we calculated separate averages for each combination of distractor location, target location, and room. Difference scores were then computed for each distractor location by subtracting the average for distractor absent trials featuring the same target location in the same room. Trials were then grouped into distractor types according to the categories defined above. For example, Near.Side distractor effects were calculated by taking the difference in average performance, for each room, between distractor absent trials in which the target was at the top location and distractor present trials in which the target was at the top location and the distractor was at the left or right location. Average trial counts, per participant, for each distractor type in each room are given in Table 4 below.

**Table 4:** Mean trials per participant for calculation of distractor type differences

	Distractor Type					
	Near.Side		Near.Top		Far	
	*Distractor-Left* *Target-Top*	*Distractor-Right* *Target-Top*	*Distractor-Top* *Target-Left*	*Distractor-Top* *Target-Right*	*Distractor-Right* *Target-Left*	*Distractor-Left* *Target-Right*
Sparse Room						
* Distractor Present*	11.0 ± 1.24 *(6–12)*	11.1 ± 1.02 *(8–12)*	10.8 ± 1.16 *(8–12)*	10.9 ± 1.29 *(7–12)*	11.3 ± 0.89 *(8–12)*	11.4 ± 0.81 *(9–12)*
* Distractor Absent*	22.5 ± 1.44 *(19–24)*	22.5 ± 1.44 *(19–24)*	22.3 ± 1.61 *(18–24)*	22.3 ± 1.86 *(15–24)*	22.3 ± 1.61 *(18–24)*	22.3 ± 1.86 *(15–24)*
Rich Room						
* Distractor Present*	10.9 ± 1.07 *(8–12)*	10.8 ± 1.04 *(8–12)*	10.8 ± 1.10 *(8–12)*	11.2 ± 0.99 *(7–12)*	10.3 ± 0.81 *(9–12)*	10.9 ± 1.28 *(6–12)*
* Distractor Absent*	22.2 ± 1.79 *(16–24)*	22.2 ± 1.79 *(16–24)*	22.2 ± 1.23 *(19–24)*	22.0 ± 1.85 *(15–24)*	22.2 ± 1.23 *(19–24)*	22.0 ± 1.85 *(15–24)*

Cells display the mean, standard deviation of the mean, and range (minimum–maximum) of trials, per participant, in each room for each combination of target and distractor locations. For each distractor type column, the counts given next to “Distractor Present” represent trials with the specified distractor location, while counts listed underneath these values, next to “Distractor Absent”, represent the distractor-absent reference trials against which the distractor-present group was compared. Final difference scores were averaged between distractor-present trials for the same distractor type, within each room

We then conducted a series of repeated-measures ANOVAs featuring room (rich, sparse) and distractor type (Near.Side, Near.Top, Far) as within-subject factors. Note that the None distractor type was not featured in these ANOVAs because each of the measures was calculated using a difference score that featured the distractor-absent trials. To control family-wise error rate, Holm–Bonferroni corrections were applied separately within each effect (distractor type, room type, distractor type × room type), treating the nine dependent variables as a family (α =.05 per family). If a main effect of distractor type was not observed in an ANOVA, we conducted a one-sample *t* test comparing the average score across all three distractor types to zero. If a main effect of distractor type was observed, we conducted separate one-sample *t* tests comparing each distractor effect to zero, with Holm–Bonferroni corrections applied to account for the three tests conducted. Similarly, we conducted paired-samples *t* tests to compare the distractor types, with Holm-Bonferroni corrections applied to account for the three comparisons performed. All reported *p* values are adjusted to reflect the Holm-Bonferroni corrections. Greenhouse–Geisser corrections were applied to all tests that violated the assumption of sphericity. Any main effects not explicitly reported failed to reach significance. Finally, we conducted exploratory cluster-based permutation testing analyses to identify the time points exhibiting significant attraction to the target, distractor, and the other (non-target, non-distractor) locations for each of the distractor types. We applied this approach because cluster-based analyses provide a simple way to identify time periods where there are sustained differences across conditions while controlling for multiple comparisons, and these approaches have been used previously in both reach-tracking (e.g., Moher et al., 2015) and EEG (e.g., Maris & Oostenveld, 2007; McNair et al., 2025) data analyses.

#### Distractor type effects

**Fig. 3:**
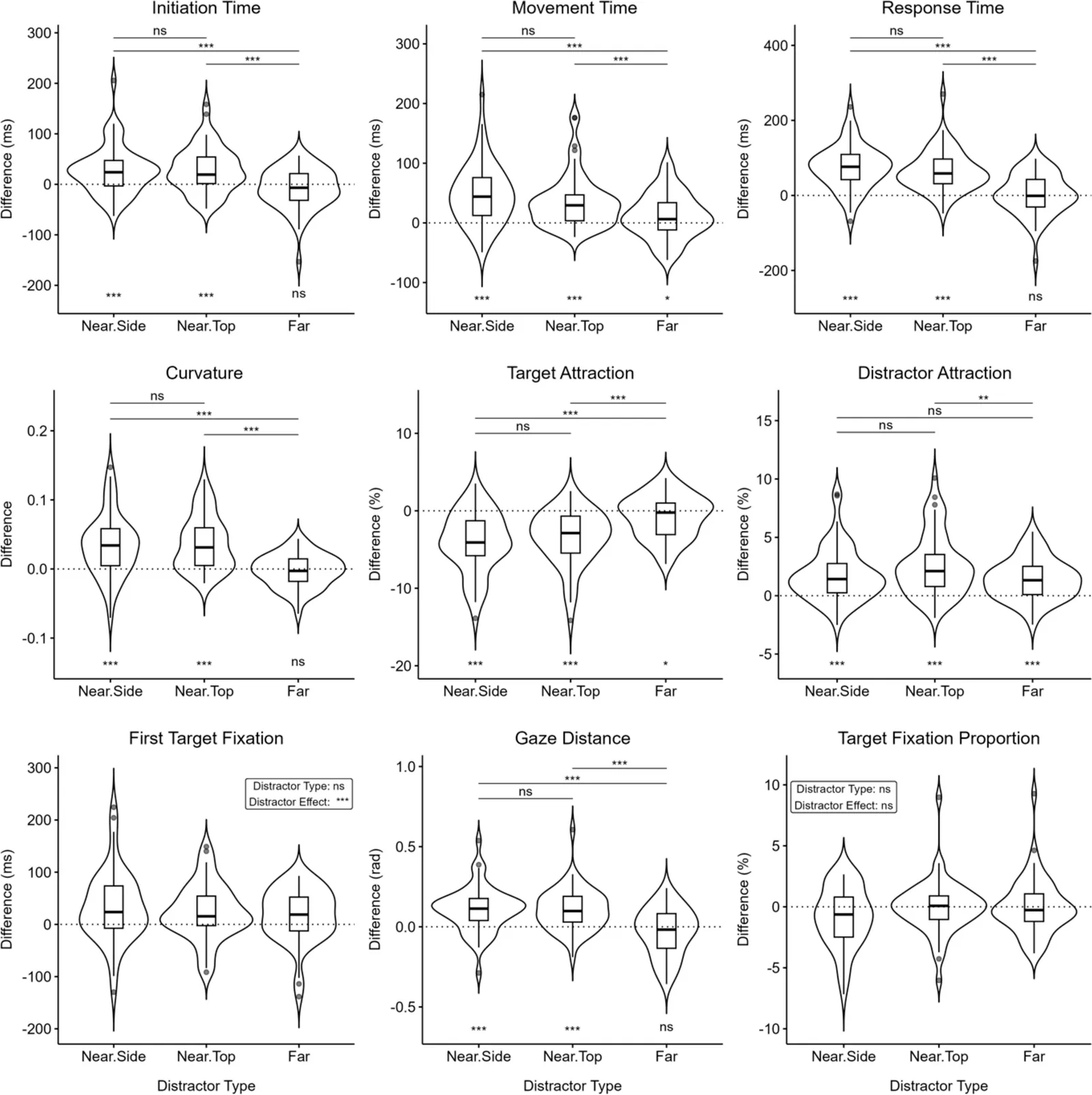
Distractor effects across distractor types for all measures. *Note.* Distractor effects reflect differences in performance between distractor present trials and distractor absent trials. Significance levels presented at the top of each panel represent comparisons between distractor types (Near.Side, Near.Top, Far), while significance levels presented at the bottom of each panel reflect comparisons to zero. In the absence of a significant interaction between distractor effect and distractor type, the significance levels of main effects are reported in a legend within individual panels. All *p* values are adjusted, as outlined in the Exploratory Analyses section. * *p* < .05, ** *p* < .01, *** *p* < .001

Average performance across each of the four distractor types is presented in Table 5 for each of the dependent measures, aside from FTF and TFP, which failed to show a significant main effect of distractor type. Consequently, we present a single difference score value for these measures reflecting the average across all trials with a distractor in the “Distractor Present” row. The effects of distractor type for each dependent measure are presented in Fig. 3, with the outcomes of pairwise comparisons presented at the top of each panel and the outcomes of one-sample *t* tests comparing scores to zero presented at the bottom of each panel. For a full written description of these results, see Section 3 of the Supplementary Materials. Here, we provide a summary of the results for each measure.

**Table 5:** Differences from distractor-absent trials, separated by distractor type

Comparison	Temporal			Reach			Gaze		
	IT	MT	RT	CURV	TA	DA	FTF	GD	TFP
Distractor Type	***	***	***	***	***	*	ns	***	ns
*None*	692 ± 18 ms	499 ± 12 ms	1191 ± 21 ms	0.119 ±.005	92.4 ± 0.58%	NA	594 ± 21 ms	2.17 ± 0.06 rad	31.2 ± 0.87%
*Near.Side*	+ 27.8 ± 6.5 ms ***	+ 47.5 ± 7.0 ms ***	+ 75.3 ± 8.1 ms ***	+ 0.034 ±.006 ***	−4.00 ± 0.53% ***	1.78 ± 0.28% ***		+ 0.110 ±.020 rad ***	
*Near.Top*	+ 29.4 ± 6.1 ms ***	+ 35.2 ± 6.0 ms ***	+ 64.6 ± 7.5 ms ***	+ 0.037 ±.005 ***	−3.44 ± 0.48% ***	2.60 ± 0.37% ***		+ 0.108 ±.018 rad ***	
*Far*	−8.4 ± 5.5 ms ns	+ 11.3 ± 5.1 ms *	+ 2.9 ± 7.3 ms ns	−0.003 ±.003 ns	−0.76 ± 0.37% *	1.38 ± 0.24% ***		−0.032 ±.021 rad ns	
Distractor Present^a^							+ 25.0 ± 5.28 ms ***		– 0.29 ± 0.24% ns

Each cell displays the mean and the standard error of the mean for each dependent variable: initiation time (IT), movement time (MT), response time (RT), reach curvature (CURV), target attraction (TA), distractor attraction (DA), first target fixation (FTF), gaze distance (GD), and target fixation proportion (TFP). The significance of each distractor type is presented in the Distractor Type row. The significance of tests comparing the observed values to 0 (indicating no distractor effect) is presented under each difference score. NA denotes that the measure was not applicable given that a distractor attraction score cannot be calculated for distractor-absent trials. *p < .05, **p < .01, ***p < .001

^a^FTF and TFP differences did not reveal a main effect of distractor type and are therefore presented with one difference score value reflecting the average across all trials with a distractor to avoid confusion regarding comparisons to 0

IT, MT, and RT all revealed main effects of distractor type (*p* values < .001). Near.Side and Near.Top distractor types differed significantly from zero for all three measures (*p* values < .001), whereas scores for the Far distractor type differed significantly from zero for MT (*p* = .032) alone. Pairwise comparisons failed to detect significant differences between the Near.Side and Near.Top distractor types for IT, MT, and RT, but did reveal significant differences between the Near.Side and Far distractor types (*p* values < .001) and between the Near.Top and Far distractor types (*p* values < .001).

CURV, TA, and DA all revealed main effects of distractor type (*p* values < .05). Near.Side and Near.Top distractor types differed significantly from zero for all three measures (*p* values < .001), whereas scores for the Far distractor type differed significantly from zero for TA (*p* = .047) and DA (*p* < .001) but not CURV. Pairwise comparisons failed to detect significant differences between the Near.Side and Near.Top distractor types for any of these measures but did reveal a significant difference between the Near.Top and Far distractor types (*p* values < .01) for each of the three measures. CURV and TA also revealed a significant difference between the Near.Side and Far distractor types (*p* values < .001), whereas DA did not.

FTF did not reveal a main effect of distractor type, but the measure did reveal a significant distractor effect, with an average distractor effect significantly above zero (*p* < .001). GD revealed a significant main effect of distractor type (*p* < .001), with scores significantly above zero for Near.Side and Near.Top distractor types (*p* values < .001) but not the Far distractor type. Pairwise comparisons revealed a significant difference between Near.Side and Far (*p* < .001) and between Near.Top and Far (*p* < .001) but not between Near.Side and Near.Top. Finally, FTP did not reveal a significant main effect of distractor type and the average distractor effect did not differ significantly from zero.

#### Room and room × distractor type effects

Our results did not reveal any significant main effects of room type or any significant interactions between room type and distractor (*p* values > .70). We again supplemented our ANOVA analyses with Bayes factors, using Faulkenberry’s (2022) *PsyStat* application. For the effect of room type, five measures revealed moderate evidence for the null (IT: BF_01_ = 4.17; RT: BF_01_ = 4.67; DA: BF_01_ = 3.74; FTF: BF_01_ = 3.40; TFP: BF_01_ = 4.23), while the remaining measures revealed anecdotal evidence for the null (MT: BF_01_ = 1.38; CURV: BF_01_ = 1.67; TA: BF_01_ = 2.20; GD: BF_01_ = 1.27). For the interaction of distractor type and room type, four measures revealed moderate evidence for the null (CURV: BF_01_ = 4.42; FTF: BF_01_ = 5.02; GD: BF_01_ = 9.38; FTP: BF_01_ = 4.15), while the remaining measures revealed strong to very strong evidence of the null (IT: BF_01_ = 28.43; MT: BF_01_ = 20.54; RT: BF_01_ = 18.96; TA: BF_01_ = 19.95; DA: BF_01_ = 50.89).

#### Attraction timeseries

For each of the final 100 timepoints in the normalized reach trajectories (excluding the first timepoint as no movement had yet happened), average target attraction scores (TAs) were calculated for each participant by taking the proportion of normalized trajectories where that index exhibited attraction towards the target. Similarly, distractor attraction scores (DAs) and attraction scores to the other (non-target, non-distractor) location (OAs) were calculated for each timepoint, per participant. For each participant, attraction difference scores for TAs, DAs, and OAs were calculated at each timepoint, for each target and distractor combination, against the average attraction on distractor-absent trials with the same target location.

**Fig. 4:**
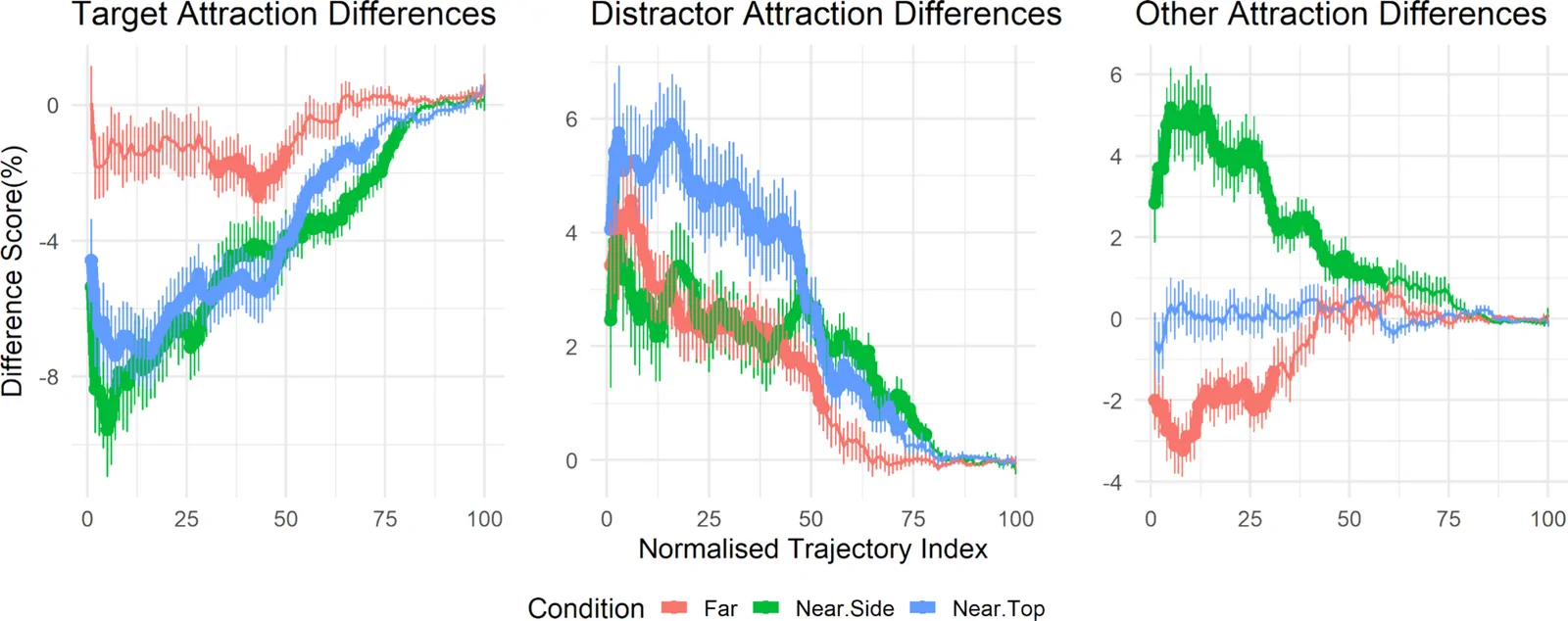
Target, distractor, and other object attraction differences for each distractor type. *Note.* Each plot shows the proportion of different attraction scores at each timepoint of the normalized reach trajectories, compared to trials with no distractor. Timepoints differing significantly from 0 are presented in thick lines. Error bars show standard errors

Cluster-based permutation analyses were then conducted on these difference scores. For each distractor type (Near.Side, Near.Top, Far), one-sample *t* tests against zero were performed at each timepoint. Clusters were defined as consecutive timepoints where the *t*-statistic exceeded the critical threshold for α =.05 (two-tailed, degrees of freedom = *N*–1) and were defined separately for positive and negative *t* values. The mass of each observed cluster was calculated as the sum of the absolute *t* values within that cluster. To assess significance, a null distribution was generated by randomly flipping the sign of the difference scores for each participant 1000 times (Nichols & Holmes, 2002; Winkler et al., 2014). For each permutation, the mass of the largest resulting cluster (based on absolute *t* values, irrespective of sign) was recorded to form the null distribution. An observed cluster was considered significant if fewer than 5% of permutation-derived maximum cluster masses exceeded its mass (*p* <.05). Significant clusters are reported as periods of attraction significantly different from the distractor-absent baseline, in either direction (Fig. 4).

Clustering permutations for attraction score differences showed that with Near.Side distractors, TAs were significantly lower from 1 to 79% of the reach trajectory (*p* < .001, *Mass* = 434), DAs were significantly higher from 1 to 78% of the reach trajectory (*p* < .001*, Mass* = 302), and OAs were significantly higher from 1 to 58% of the reach trajectory (*p* < .001, *Mass* = 252), compared to distractor-absent reach attraction scores. This suggests that when the target was at the top location and the distractor was at the left or right location, reach trajectories exhibited attraction to either the distractor or the other (non-target, non-distractor) object, with the latter possibly reflecting repulsion from the distractor on a subset of trials (e.g., Erb et al., 2016). Consequently, attraction towards the target decreased for the majority of the reach trajectory, before the response was ultimately corrected to the target location.

For trials with Near.Top distractors, clustering permutations showed that TAs were significantly lower from 1 to 72% of the reach trajectory (*p* < .001 *Mass* = 381), DAs were significantly higher from 1 to 72% of the reach trajectory (*p* < .001*, Mass* = 349), and OAs showed no significant clusters, compared to distractor-absent reach attraction scores. This suggests that when the target was at the left or right location, and the distractor is at the top location, reach trajectories were initiated towards the distractor. Consequently, distractor attraction increased, and target attraction decreased for the majority of the reach movement, before the response was ultimately corrected to the target location.

For trials with Far distractors, clustering permutations showed that TAs were significantly lower from 32 to 50% of the reach trajectory (*p* < .05 *Mass* = 50), DAs were significantly higher from 1 to 53% of the reach trajectory (*p* < .001*, Mass* = 234), and OAs were significantly lower from 1 to 31% of the reach trajectory (*p* < .01*, Mass* = 109), compared to distractor-absent reach attraction scores. This suggests that when the target was at the left or right location, and the distractor was at the opposite location, reach trajectories were initiated towards the distractor, increasing distractor attraction, before ultimately being corrected towards the target location. However, compared to distractor-absent trials, target attraction was not significantly affected for the first 32% of the reach trajectory, while attraction towards the other (non-target, non-distractor) object was significantly lower for the first 31% of the reach trajectory. As the other (non-target, non-distractor) object was always at the top location on Far distractor trials, these cluster differences suggest that on distractor-absent trials, responses may have been initiated towards the top location, before quickly being redirected towards the target location. When a distractor was present on the opposite side from the target, responses were more likely to be initiated to the distractor, resulting in a relative decrease in attraction to the other object early in the movement and a decrease in attraction toward the target later in the movement.

## Discussion

The present study investigated the dynamics of attentional capture in an additional singleton task by using a virtual reality (VR) system to simultaneously measure participants’ hand and eye movements. Across temporal, reach, and gaze measures, our results provided (1) convergent evidence for robust capture effects in VR, (2) demonstrated the utility of continuous target and distractor attraction measures of spatial trajectories, and (3) indicated that the richness of the virtual environment impacted some general measures of performance but had no statistically significant impact on the size of attentional capture costs. We discuss each of these findings in turn in the following sections.

### Dynamics of attentional capture in 3D environments

The results of our planned analyses revealed robust attentional capture costs across our temporal, reach, and gaze measures. Relative to distractor-absent trials, distractor-present trials featured significantly slower initiation times (ITs), movement times (MTs), and response times (RTs), larger reach curvatures (CURVs) and distractor attraction scores (DAs), lower target attraction scores (TAs), slower first target fixation latencies (FTFs), and larger gaze distance scores (GDs). Overall, these effects are consistent with a large body of research that has used button-press, hand-tracking, and eye-tracking methods to investigate attentional capture with two-dimensional visual stimuli. Demonstrating that such attentional capture effects generalize to three-dimensional environments is an important step toward bridging the gap between laboratory paradigms and our everyday experiences in the real world.

Our exploratory analyses investigating target-distractor proximity further reinforce the generalizability of attentional capture findings from two- to three-dimensional visual environments. In light of previous work indicating that attentional capture costs can be impacted by the degree of distance separating the target and distractor (e.g., Gaspar & McDonald, 2014; Hickey & Theeuwes, 2011), we conducted exploratory analyses that examined capture costs across trials in which the target was in the top location and the distractor was at the left or right location (Near.Side trials), trials in which the target was at the left or right location and the distractor was at the top location (Near.Top trials), and trials in which the target was at either the left or right side and the distractor was at the opposite location (Far trials). Each of our measures aside from TFP revealed significant attentional capture costs on both Near.Side and Near.Top trials,^3^ with none of the measures revealing significant differences between the two distractor types. On Far trials, significant attentional capture effects were observed in MT, TA, and DA, with significantly smaller effects in Far trials than in Near.Side and/or Near.Top trials for MT, TA, and DA, and a significant overall distractor effect in FTF regardless of distractor type. These findings are broadly consistent with previous button-press investigations of the additional singleton task featuring circular arrays that have observed decreasing attentional capture costs as target-distractor distance increases (e.g., Gaspar & McDonald, 2014; Hickey & Theeuwes, 2011).

However, two results deviated at least somewhat from the general trend of increasing target-distractor distances corresponding to decreasing attentional capture costs. DA difference scores were significantly larger on Near.Top than Far trials but did not differ significantly between Far trials and Near.Side trials, indicating that distractors located at the left and right locations attracted hand movements to comparable degrees, regardless of whether the target was relatively near or far. Additionally, FTF difference scores were equivalent across the three distractor types, indicating that distractors resulted in participants taking longer to fixate on the target, even when the target and distractor were presented in opposite hemifields.

Performance in the current study could have been impacted by differences in how visual information is processed along the horizontal and vertical meridians. Previous research has consistently demonstrated that visual information is processed more efficiently along the horizontal than the vertical meridian (e.g., Carrasco et al., 2004), likely due in part to the anatomical structure of the visual system (e.g., Kupers et al., 2022; Lee & Carrasco, 2025). A recent study also found that salient distractors along the horizontal meridian produced greater interference than distractors along the vertical meridian (Kerzel & Constant, 2024). The current study did not reveal robust evidence of such an effect, with the effect of the distractor not differing significantly between the Near.Side trials (distractor on the horizontal meridian) and the Near.Top trials (distractor on the vertical meridian). To further explore these null effects, we computed Bayes factors for the paired-samples *t* tests comparing Near.Side and Near.Top trials (see Section 3 of the Supplementary Materials). Bayesian approximations revealed moderate evidence for the null effect in IT, RT, CURV, TA, and GD (BF_01_ values ranging from 4.24 to 6.65), anecdotal evidence for the null effect in MT (BF_01_ = 2.16), and anecdotal evidence for the alternative hypothesis in DA (BF_10_ = 1.19). Thus, the evidence for the null is somewhat mixed and differs across our measures.

The apparent discrepancy between our results and those of Kerzel and Constant (2024) could have stemmed from a range of methodological differences between the studies. For example, the current study featured a relatively limited number of locations at which the target or distractor could appear. In contrast to Kerzel and Constant (2024), participants in the current study were not instructed to maintain fixation at a centrally presented stimulus, which allowed participants to adopt different visual search strategies. Additionally, the use of reaching methods may complicate the examination of meridian effects (e.g., Doumen et al., 2006). We therefore do not make any strong claims about meridian effects in the current study and believe that future studies designed to investigate meridian effects in greater detail are required.

In summary, these findings demonstrate that VR presents a robust method for simultaneously capturing the manual and oculomotor dynamics of attentional capture in 3D environments. This approach to investigating attentional capture introduces new opportunities to examine hand–eye coordination, individual differences in search strategies, the links between motoric costs and attentional capture (e.g., Anderson et al., 2025), and depth and field of view manipulations.

### Measuring capture in 3D

Researchers using three-dimensional hand-tracking techniques to investigate visual attention often use a two-dimensional projection of the full three-dimensional data to calculate measures such as curvature, maximum perpendicular deviation, or area under the curve (e.g., Erb et al., 2022; Moher et al., 2015; Welsh, 2011). This approach has proven effective for capturing effects that may otherwise be obscured in temporal measures like RT. However, such approaches provide limited insight into how competition between response alternatives unfolds across time. For example, larger reach curvatures do not necessarily indicate that a competing response was activated. Increased curvatures can also reflect delayed decision making, as participants will often initiate a movement toward the center of the display before deciding on a particular response. Consequently, researchers frequently need to supplement curvature results with change-of-mind measures (Erb & Marcovitch, 2019) or attraction scores (Erb et al., 2016) to demonstrate if and when a competing response was activated.

In contrast to reach curvatures, our three-dimensional target and distractor attraction scores provided robust continuous measures of performance. Although CURV, TA, and DA all revealed significant capture costs in our primary analyses, TA and DA offered more robust and directly interpretable measures of performance. Additionally, DA difference scores detected a large, significant effect of attentional capture on Far distractor trials that was non-significant in CURV difference scores. To determine whether this discrepancy between CURV and DA emerged because CURV was calculated using two-dimensional projections whereas DA was calculated using three-dimensional trajectories, we recalculated TA and DA scores using only the x and y dimensions and re-ran all our analyses using the two-dimensional scores. The same patterns of effects were observed across both the two-dimensional and the three-dimensional TA and DA scores, with some minor differences emerging regarding the timing of effect onsets and offsets (see Section 5 of the Supplementary Materials). Consequently, the discrepancy between CURV and DA scores likely reflected the inability of CURV to distinguish between delayed decision making and the activation of a competing response rather than differences stemming from the use of two- versus three-dimensional trajectories.

Additionally, TA and DA provide continuous measures of performance that allow for the time course of effects to be examined. As our cluster-based permutation tests demonstrate, attentional capture costs in DA generally emerged from the onset of movements and remained significant for between 53 and 78% of the movement, depending on distractor type. However, our exploratory analyses also underscored the need for caution when interpreting TA and DA measures. For example, differences in the size, strength, and timing of distractor attraction effects can reflect factors related to response tendencies (e.g., a disposition to initially look at or move toward a particular response) and the length of the overall trajectory (e.g., movement trajectories on Far distractor trials will tend to be longer than on Near distractor trials, resulting in a larger proportion of the movement being directed at the target following movement redirection).

In summary, our TA and DA scores appear to provide a more sensitive and directly interpretable measure of attentional capture than CURV, allowing for the identification of the relative onset and offset of attraction effects. In contrast to other approaches that have averaged reach trajectories to identify the extent to which movements were attracted to a competitor over time (e.g., Erb et al., 2016), computing attraction scores at each time point within a movement trajectory provides a more precise measure of when a distractor ceases to exert an influence on a reach movement. This is because early attraction toward a distractor can result in movement trajectories that are non-overlapping with distractor-absent trajectories for large portions of the movement after the distractor is no longer exerting an attraction on the hand, thereby complicating the detection of the offset of the distractor’s effect.

### Environmental complexity

Overall, visual search in the current study appears to have been less efficient in the rich visual environment than in the sparse environment. Participants exhibited slower ITs, larger gaze distances, and larger target fixation proportions in the rich versus the sparse environment, indicating that overall processing demands increased with the complexity of the environment. These results are consistent with previous eye-tracking work with two-dimensional displays indicating that visual search is less efficient in cluttered environments (Semizer & Michel, 2019; Semizer & Rosenholtz, 2025). Interestingly, first target fixation times were comparable across the two environments, suggesting that participants tended to locate the target at similar latencies, even if they moved their eyes more and looked at the target for a greater proportion of the trial in the rich environment. We suspect that the increased target fixation proportions observed in the rich environment reflected an increased need to fixate on the target to effectively guide reach movements.

None of our measures revealed a significant interaction between the richness of the virtual environment and distractor presence or absence. Bayesian approximations of these null effects revealed moderate evidence for the null in IT, RT, and TFP (BF_01_ values ranging from 3.40 to 4.64) and anecdotal evidence for the null in MT, CURV, TA, FTF, and GD. Thus, the evidence that environmental richness failed to impact participants’ abilities to avoid or recover from attentional capture within the specific virtual environments tested in the current study was mixed and confined to specific measures. Further research with a larger sample size is needed to evaluate the extent to which these results hold across a wider range of visual environments, including dynamic environments and environments that afford interactions with a wider range of objects.

One important caveat to the results outlined above concerns the effect of environment order on ITs, RTs, and GDs observed in our preliminary analyses. When participants began in the rich environment, overall ITs, RTs, and GDs in the sparse environment decreased markedly, suggesting practice or adaptation effects. By contrast, ITs, RTs, and GDs were comparable across both environments when participants began in the sparse environment. This asymmetry suggests that the effect of environmental richness on ITs, RTs, and GDs was reduced or removed through practice in the sparse environment. For example, participants may have developed a better sense of spatial layout and distances in the sparse environment and then benefited from this experience when planning and executing their responses in the rich environment.

However, given that all participants completed the baseline trials in the rich environment before beginning the experimental blocks, it is possible that the observed effect of room order was driven by previous experience within the rich environment. For example, transitioning from the rich room for the baseline trials to the sparse room for the first set of experimental blocks may have served to highlight the similarities in the spatial layout of the virtual objects, resulting in better performance after returning to the rich environment for the final experimental blocks. Future research should therefore remove this potential confound by having participants complete the baseline trials in a separate environment or by counterbalancing whether participants performed the baseline trials in the rich or sparse environment.

### Limitations and future directions

One limitation of the current study is that aspects of our task design may have allowed participants to adopt a wider range of strategies than anticipated. For example, participants may have learned that the object presented in the bottom location reliably indicated the non-target shape and the target color. Such a strategy could have decreased the size of the distractor effect observed in the current study by guiding visual attention away from the distractor. Similarly, participants in our study may have learned that the saliently colored distractor always appeared in the non-target shape, which could have encouraged participants to search for the distractor to determine the shape of the target. However, our eye-tracking data suggests that participants did not routinely search for the distractor, with the majority of participants adopting a stable search strategy that involved looking to a specific location (usually, the top location) at the outset of each trial. To avoid the adoption of such strategies, future research could present participants with more complex layouts using a larger number of objects and multiple object shapes (following Wang & Theeuwes, 2018), keep the target shape consistent throughout the study (following Erb et al., 2022), or place the objects at random locations on each trial. It is also possible that participants were not in a pure singleton detection mode, as only two target shapes were used and participants thus could have activated two simultaneous target templates (e.g., Lagroix et al., 2018). We think this unlikely, as prior research using very similar tasks has shown that participants will adopt singleton detection mode when two target shapes are possible (e.g., Lamy & Egeth, 2003), and in fact there is evidence to suggest participants are not able to voluntarily adopt feature search mode when targets are always presented as singletons (e.g., Bacon & Egeth, 1994; Leber & Egeth, 2006). Nevertheless, future research could further explore the impact of target and distractor variability on search in VR contexts.

A second limitation of the current study is that our task was not specifically designed to examine target-distractor proximity effects. Future work should explore the impact of target-distractor proximity as well as the impact of presenting the target and distractor in the same hemifield versus across hemifield by using a circular array of shapes (as in Gaspar & McDonald, 2014; Hickey & Theeuwes, 2011; Wang & Theeuwes, 2020; Wang et al., 2019). Increasing the number of objects presented on each trial could also help to eliminate the consistent search strategies adopted by the participants in the current experiment, which prevented us from examining how first saccades were impacted by the presence or absence of a distractor.

Finally, while our results demonstrate the efficacy of measuring attentional capture in hand and eye behavior using VR, a wide range of analyses and modeling techniques could be applied to our data to gain insight into the processes underlying behavior. For example, while our cluster-based approach for identifying significant time periods of differences across conditions controls for multiple comparisons within a single analysis, we applied multiple versions of that analysis across the study, and other approaches may be better suited for global minimization of type I errors. Additionally, our data could prove informative for efforts to test models of hand–eye coordination in reaching tasks (Binsted et al., 2001; Jana & Murthy, 2021). Although detailed examination of such coordinative dynamics extends beyond the scope of the current study, Section 4 of the Supplementary Materials provides exploratory analyses examining correlations among our hand and eye measures. Additionally, our data sets have been made available online so that researchers can conduct more in-depth explorations.

### Practical guidance and comparisons with real-world performance

The most challenging component of completing the current study was developing software in Python to visualize and process the hand- and eye-tracking data. Ultimately, our aim is to release an open-source, user-friendly version of this software featuring a graphical user interface, detailed code annotations, and supporting documentation so that the approach can be used more widely. However, alternative software solutions are currently available, including the Gaze and Movement Assessment (GaMA) software developed at the University of Alberta (Williams et al., 2019). Notably, GaMA has recently been used to process data from a virtual reality study investigating eye-hand coordination (Lavoie et al., 2025).

Although we view the simultaneous measurement of hand and eye movements to be a particular strength of VR as a behavior research method, tools such as the Unity Experiment Framework (2.0; Brookes et al., 2020) make it relatively easy to translate traditional visual search tasks using button-press measures of performance into VR given that response time and accuracy can be recorded directly from controller inputs without requiring extensive data processing with specialized software. Additionally, a wide range of assets can be freely obtained through the Unity Asset Store (Unity Technologies, 2026), decreasing barriers associated with the construction of immersive virtual environments. Research teams looking to incorporate VR into their laboratories may therefore find the barriers to adoption to be more manageable when retaining traditional button-press measures of performance.

Research teams specifically looking to adopt VR as a method for recording continuous behavioral dynamics can find a range of helpful resources from the existing hand-tracking literature, including recommendations regarding the collection of baseline reach trajectories, the resampling of movement trajectories in space versus time, the screening of individual trials (Gallivan & Chapman, 2014), the use of single-trial analyses (Dotan et al., 2019), and the use of cluster analysis to classify trajectories (Wulff et al., 2019). It should also be noted that VR-derived movement measures will reflect a lag between when a participant moves and when that movement is presented within the headset. This lag, known as the motion-to-photon latency, is impacted by the specific motion prediction algorithms used by the hardware and has been reported to be between 21 and 42 ms for movements featuring sudden onsets and between 2 and 13 ms for ongoing movements along a single plane (Warburton et al., 2023). Although this latency does not inherently complicate comparisons among movements within a VR task (e.g., comparing ITs on distractor-absent versus distractor-present trials), it would introduce differences between VR-derived movement measures and measures recorded using electromagnetic or optical tracking systems. Consequently, caution is needed when comparing the timing of effects from VR-derived movement measures to those observed with electromagnetic or optical tracking systems.

In contrast to our previous hand-tracking research using an electromagnetic tracking system (e.g., Erb et al., 2022), which allowed participants to rest their hand on a table between each trial, we have found that reaching tasks in VR tend to be more fatiguing when participants are required to hold the controller in front of them between each trial. We provided frequent breaks in the current study to manage potential fatigue. However, VR hand-tracking tasks could be designed to allow participants to rest their hands between trials, particularly as controller-free hand-tracking improves.

Researchers interested in using VR headsets to record eye movements should consider whether the accuracy and sampling rate provided by the headset are suitable for the research question under consideration. Professional screen-mounted eye tracking systems typically achieve an accuracy of between 0.2° and 0.5° of visual angle with sampling rates up to 1200 Hz, whereas the Vive Pro Eye headset used in the current study has been found to generate an average binocular accuracy of 1.08° (Schuetz & Fiehler, 2022) and a maximum sampling rate of 120 Hz. Although the accuracy and sampling rate of the Vive Pro Eye headset were appropriate for the current study, the eye tracking afforded by VR headsets may not be suitable for studies requiring more fine-grained measures of looking behavior. Conversely, an advantage of eye tracking paradigms within VR is the ability to present all participants with the same viewing conditions within an immersive environment regardless of their height, posture, and seating conditions. In the present investigation, each participant’s eye level was recorded at the start of the instruction sequence. The experimental display was then adjusted to this height, ensuring that stimuli appeared at the same relative position for each participant. The precise control over viewing conditions afforded by VR could facilitate paradigms with stricter view requirements by, for example, locking a stimulus array to the head or gaze direction to ensure stimuli remain in peripheral vision.

However, caution is needed when attempting to draw connections between behavior observed in VR studies and behavior observed in the real world. Gaze behaviors in VR environments and physical environments may differ in consequential ways given that modern VR headsets induce a sense of depth through stereoscopic cues while disrupting the natural coupling between vergence and accommodation (Arefin et al., 2025; Pastel et al., 2021; Wang et al., 2025). For example, Arefin et al. (2025) observed small but significant differences in eye vergence angles across real-world, augmented reality (AR), and VR conditions. Similarly, in the context of manual pointing behavior, Wang et al. (2025) found that movements in the real-world were faster than movements in VR but equally variable, exhibiting comparable levels of online control. Although these studies indicate that the differences between the behaviors observed in the real world and VR can often be relatively small, more research exploring a wider range of tasks and conditions is needed to identify the extent to which the patterns observed in VR might differ from those observed in the real world.

## Conclusion

This study demonstrates the efficacy of a practical methodological framework for investigating the dynamics of attentional control in hand and eye movements within immersive virtual environments. In addition to revealing robust attentional capture costs in hand and eye measures in a three-dimensional environment, our results underscore the value of calculating target and distractor attraction scores when examining the nature and time course of attentional capture. Given that VR systems offer a relatively affordable and portable method for measuring hand and eye movements, this approach can be readily adopted by researchers to examine attention, perception, and action in controlled yet ecologically rich settings. VR is therefore well placed to bridge the gap between traditional laboratory paradigms and everyday experiences.

## Supplementary Information

Below is the link to the electronic supplementary material.

ESM 1 Supplementary Material 1 (DOCX 530 KB)

## Footnotes

1 Although not specifically focused on attentional capture, a number of studies have investigated visual attention with simultaneous measures of hand and eye movements, including Khan et al. (2011), Moher and Song (2016), Song and McPeek (2009).

2 As previously noted, the visual angles for the bottom location and the starting orb were similar. Consequently, it is possible that some of the participants classified as adopting a search strategy prioritizing the bottom location may have been beginning trials by looking at the starting orb.

3 Technically, FTF did not show a main effect of distractor type, so Near.Side and Near.Top were not examined separately, but the measure showed a strong overall distractor effect.

## Declarations

### Open practices statement

The analyses presented in this manuscript were not pre-registered. The data, analysis scripts, task materials, and supplementary materials for this project are available at https://osf.io/hcz4m and https://github.com/CMND-Lab/VR-SingletonCapture_ViveProEye_OSF.
