# Supplementary material for: Measuring the dynamics of attentional capture in hand and eye movements with virtual reality
Source: Behav Res Methods. 2026 Sep 24;58(11):302. doi: 10.3758/s13428-026-03177-9 (PMC13612694; doi:10.3758/s13428-026-03177-9)
Supplement: Supplementary file 1 [file 13428_2026_3177_MOESM1_ESM.docx]

# Supplementary Materials

## Section 1: Preliminary Analysis

The secondary factors examined in the preliminary ANOVAs are room counterbalance (Real-Blank, Blank-Real), target shape (Apple, Pear), target color (Green, Red), and target location (left, top, right). For each of these secondary factors, an ANOVA was conducted along with the primary factors of room (Real, Blank) and distractor presence (Present, Absent), with Greenhouse-Geisser correction applied if the comparison group violated assumptions of sphericity. To control family-wise error rate, Holm-Bonferroni corrections were applied separately within each interaction effect featuring a secondary factor and a primary factor (secondary factor × distractor presence, secondary factor × room, secondary factor × distractor presence × room), treating the 8 dependent variables (initiation time, movement time, response time, curvature, target attraction, first target fixation, gaze distance, target fixation proportion) as a family for each effect (α = .05 per family). Note that DA was excluded from these analyses because it was calculated as a difference score between distractor present and distractor absent trials in a manner that controlled for room and target location.

Effects that remained significant following Holm-Bonferroni correction were further investigated using follow-up *t*-tests. Given that the counterbalance factor varied between participants, independent samples *t*-tests were used to examine these effects, while paired samples *t*-tests were used for the remaining within-subjects effects. To control the familywise error rate, Holm-Bonferroni corrections were applied separately to the follow-up *t*-tests for each factor, treating each factor's comparisons as a distinct family (e.g., follow-up tests examining target location effects were corrected to reflect the three comparisons performed).

### Counterbalance

**Counterbalance × Room**

| Effect | DFn | DFd | F | p | <.05 | pes | p_holm | <.05 |
| --- | --- | --- | --- | --- | --- | --- | --- | --- |
| initiation.time | 1 | 52 | 11.573 | 0.001000 | ** | 0.182 | 0.006000 | ** |
| movement.time | 1 | 52 | 1.094 | 0.300000 | ns | 0.021 | 1.000000 | ns |
| response.time | 1 | 52 | 13.647 | 0.000531 | *** | 0.208 | 0.003717 | ** |
| curvature | 1 | 52 | 0.003 | 0.957000 | ns | 0.000 | 1.000000 | ns |
| target.attraction | 1 | 52 | 0.188 | 0.666000 | ns | 0.004 | 1.000000 | ns |
| first.target.fixation | 1 | 49 | 0.041 | 0.840000 | ns | 0.001 | 1.000000 | ns |
| gaze.distance | 1 | 49 | 22.489 | 0.000019 | *** | 0.315 | 0.000149 | *** |
| target.fixation.proportion | 1 | 49 | 0.438 | 0.511000 | ns | 0.009 | 1.000000 | ns |

Initiation Time - Counterbalance × Room

| Room comparison by Counterbalance: Paired *t* tests  *alternative hypothesis: true mean difference is not equal to 0* | | | | | | | | |  |
| --- | --- | --- | --- | --- | --- | --- | --- | --- | --- |
| counterbalance | group1 | group2 | DF | statistic | p | p<.05 | p_holm | <.05 |  |
| Blank-Real | First | Second | 26 | -0.4503067 | 0.65622182 | ns | 0.65622182 | ns |  |
| Real-Blank | First | Second | 26 | 4.9429429 | 0.00003907 | *** | 0.00007814 | *** |  |
| Counterbalance comparison by Room: Unpaired *t* tests  *alternative hypothesis: true mean difference is not equal to 0* | | | | | | | | |  |
| room.order | group1 | group2 | DF | statistic | p | p<.05 | p_holm | <.05 |  |
| First | Real-Blank | Blank-Real | 50.47106 | 1.251441 | 0.2165430 | ns | 0.4330861 | ns |  |
| Second | Real-Blank | Blank-Real | 50.51328 | -1.088512 | 0.2815352 | ns | 0.4330861 | ns |  |

Response Time - Counterbalance × Room

| Room comparison by Counterbalance: Paired *t* tests  *alternative hypothesis: true mean difference is not equal to 0* | | | | | | | | |  |
| --- | --- | --- | --- | --- | --- | --- | --- | --- | --- |
| counterbalance | group1 | group2 | DF | statistic | p | p<.05 | p_holm | <.05 |  |
| Blank-Real | First | Second | 26 | -0.570927 | 0.57295131 | ns | 0.57295131 | ns |  |
| Real-Blank | First | Second | 26 | 5.881982 | 0.00000334 | *** | 0.00000668 | *** |  |
| Counterbalance comparison by Room: Unpaired *t* tests  *alternative hypothesis: true mean difference is not equal to 0* | | | | | | | | |  |
| room.order | group1 | group2 | DF | statistic | p | p<.05 | p_holm | <.05 |  |
| First | Real-Blank | Blank-Real | 49.64093 | 0.7944965 | 0.43068851 | ns | 0.4306885 | ns |  |
| Second | Real-Blank | Blank-Real | 50.75799 | -1.7606570 | 0.08432048 | ns | 0.1686410 | ns |  |

Gaze Distance - Counterbalance × Room

| Room comparison by Counterbalance: Paired *t* tests  *alternative hypothesis: true mean difference is not equal to 0* | | | | | | | | |  |
| --- | --- | --- | --- | --- | --- | --- | --- | --- | --- |
| counterbalance | group1 | group2 | DF | statistic | p | p<.05 | p_holm | <.05 |  |
| Blank-Real | First | Second | 25 | -0.402760 | 0.69054824 | ns | 0.69054824 | ns |  |
| Real-Blank | First | Second | 24 | 6.673153 | 0.00000067 | *** | 0.00000134 | *** |  |
| Counterbalance comparison by Room: Unpaired *t* tests  *alternative hypothesis: true mean difference is not equal to 0* | | | | | | | | |  |
| room.order | group1 | group2 | DF | statistic | p | p<.05 | p_holm | <.05 |  |
| First | Real-Blank | Blank-Real | 48.60343 | 1.810628 | 0.07638198 | ns | 0.1013971 | ns |  |
| Second | Real-Blank | Blank-Real | 47.61913 | -2.004704 | 0.05069853 | ns | 0.1013971 | ns |  |

**Counterbalance × Distractor**

| Effect | DFn | DFd | F | p | <.05 | pes | p_holm | <.05 |
| --- | --- | --- | --- | --- | --- | --- | --- | --- |
| initiation.time | 1 | 52 | 1.227 | 0.273000 | ns | 0.023 | 1.000000 | ns |
| movement.time | 1 | 52 | 0.633 | 0.430000 | ns | 0.012 | 1.000000 | ns |
| response.time | 1 | 52 | 2.33 | 0.133000 | ns | 0.043 | 1.000000 | ns |
| curvature | 1 | 52 | 0.25 | 0.619000 | ns | 0.005 | 1.000000 | ns |
| target.attraction | 1 | 52 | 0.085 | 0.771000 | ns | 0.002 | 1.000000 | ns |
| first.target.fixation | 1 | 49 | 1.165 | 0.286000 | ns | 0.023 | 1.000000 | ns |
| gaze.distance | 1 | 49 | 0.73 | 0.397000 | ns | 0.015 | 1.000000 | ns |
| target.fixation.proportion | 1 | 49 | 0.343 | 0.561000 | ns | 0.007 | 1.000000 | ns |

**Counterbalance × Room × Distractor**

| Effect | DFn | DFd | F | p | <.05 | pes | p_holm | <.05 |
| --- | --- | --- | --- | --- | --- | --- | --- | --- |
| initiation.time | 1 | 52 | 3.214 | 0.079000 | ns | 0.058 | 0.474000 | ns |
| movement.time | 1 | 52 | 1.775 | 0.189000 | ns | 0.033 | 0.756000 | ns |
| response.time | 1 | 52 | 5.997 | 0.018000 | * | 0.103 | 0.144000 | ns |
| curvature | 1 | 52 | 0.013 | 0.911000 | ns | 0.000 | 1.000000 | ns |
| target.attraction | 1 | 52 | 1.517 | 0.224000 | ns | 0.028 | 0.756000 | ns |
| first.target.fixation | 1 | 49 | 2.197 | 0.145000 | ns | 0.043 | 0.725000 | ns |
| gaze.distance | 1 | 49 | 5.27 | 0.026000 | * | 0.097 | 0.182000 | ns |
| target.fixation.proportion | 1 | 49 | 0.012 | 0.914000 | ns | 0.000 | 1.000000 | ns |

### Target Shape

**Target Shape × Room**

| Effect | DFn | DFd | F | p | <.05 | pes | p_holm | <.05 |
| --- | --- | --- | --- | --- | --- | --- | --- | --- |
| initiation.time | 1 | 53 | 0.045 | 0.833000 | ns | 0.001 | 1.000000 | ns |
| movement.time | 1 | 53 | 2.014 | 0.162000 | ns | 0.037 | 0.972000 | ns |
| response.time | 1 | 53 | 0.69 | 0.410000 | ns | 0.013 | 1.000000 | ns |
| curvature | 1 | 53 | 5.47 | 0.023000 | * | 0.094 | 0.184000 | ns |
| target.attraction | 1 | 53 | 1.813 | 0.184000 | ns | 0.033 | 0.972000 | ns |
| first.target.fixation | 1 | 50 | 1.014 | 0.319000 | ns | 0.020 | 1.000000 | ns |
| gaze.distance | 1 | 50 | 0.327 | 0.570000 | ns | 0.007 | 1.000000 | ns |
| target.fixation.proportion | 1 | 50 | 3.07 | 0.086000 | ns | 0.058 | 0.602000 | ns |

**Target Shape × Distractor**

| Effect | DFn | DFd | F | p | <.05 | pes | p_holm | <.05 |
| --- | --- | --- | --- | --- | --- | --- | --- | --- |
| initiation.time | 1 | 53 | 0.037 | 0.847000 | ns | 0.001 | 1.000000 | ns |
| movement.time | 1 | 53 | 0.083 | 0.774000 | ns | 0.002 | 1.000000 | ns |
| response.time | 1 | 53 | 0.007 | 0.934000 | ns | 0.000 | 1.000000 | ns |
| curvature | 1 | 53 | 7.884 | 0.007000 | ** | 0.129 | 0.056000 | ns |
| target.attraction | 1 | 53 | 3.606 | 0.063000 | ns | 0.064 | 0.441000 | ns |
| first.target.fixation | 1 | 50 | 1.991 | 0.164000 | ns | 0.038 | 0.820000 | ns |
| gaze.distance | 1 | 50 | 0.042 | 0.838000 | ns | 0.001 | 1.000000 | ns |
| target.fixation.proportion | 1 | 50 | 2.953 | 0.092000 | ns | 0.056 | 0.552000 | ns |

**Target Shape × Room × Distractor**

| Effect | DFn | DFd | F | p | <.05 | pes | p_holm | <.05 |
| --- | --- | --- | --- | --- | --- | --- | --- | --- |
| initiation.time | 1 | 53 | 0.993 | 0.323000 | ns | 0.018 | 1.000000 | ns |
| movement.time | 1 | 53 | 1.366 | 0.248000 | ns | 0.025 | 1.000000 | ns |
| response.time | 1 | 53 | 1.959 | 0.167000 | ns | 0.036 | 1.000000 | ns |
| curvature | 1 | 53 | 1.508 | 0.225000 | ns | 0.028 | 1.000000 | ns |
| target.attraction | 1 | 53 | 0.951 | 0.334000 | ns | 0.018 | 1.000000 | ns |
| first.target.fixation | 1 | 50 | 2.624 | 0.112000 | ns | 0.050 | 0.896000 | ns |
| gaze.distance | 1 | 50 | 0.516 | 0.476000 | ns | 0.010 | 1.000000 | ns |
| target.fixation.proportion | 1 | 50 | 0.432 | 0.514000 | ns | 0.009 | 1.000000 | ns |

### Target Colour

**Target Colour × Room**

| Effect | DFn | DFd | F | p | <.05 | pes | p_holm | <.05 |
| --- | --- | --- | --- | --- | --- | --- | --- | --- |
| initiation.time | 1 | 53 | 0.015 | 0.903000 | ns | 0.000 | 1.000000 | ns |
| movement.time | 1 | 53 | 0.087 | 0.769000 | ns | 0.002 | 1.000000 | ns |
| response.time | 1 | 53 | 0.098 | 0.755000 | ns | 0.002 | 1.000000 | ns |
| curvature | 1 | 53 | 2.745 | 0.103000 | ns | 0.049 | 0.824000 | ns |
| target.attraction | 1 | 53 | 0.752 | 0.390000 | ns | 0.014 | 1.000000 | ns |
| first.target.fixation | 1 | 50 | 0.276 | 0.602000 | ns | 0.005 | 1.000000 | ns |
| gaze.distance | 1 | 50 | 0.392 | 0.534000 | ns | 0.008 | 1.000000 | ns |
| target.fixation.proportion | 1 | 50 | 1.101 | 0.299000 | ns | 0.022 | 1.000000 | ns |

**Target Colour × Distractor**

| Effect | DFn | DFd | F | p | <.05 | pes | p_holm | <.05 |
| --- | --- | --- | --- | --- | --- | --- | --- | --- |
| initiation.time | 1 | 53 | 6.772 | 0.012000 | * | 0.113 | 0.096000 | ns |
| movement.time | 1 | 53 | 0.838 | 0.364000 | ns | 0.016 | 1.000000 | ns |
| response.time | 1 | 53 | 1.187 | 0.281000 | ns | 0.022 | 1.000000 | ns |
| curvature | 1 | 53 | 0.762 | 0.387000 | ns | 0.014 | 1.000000 | ns |
| target.attraction | 1 | 53 | 1.055 | 0.309000 | ns | 0.020 | 1.000000 | ns |
| first.target.fixation | 1 | 50 | 0.818 | 0.370000 | ns | 0.016 | 1.000000 | ns |
| gaze.distance | 1 | 50 | 2.944 | 0.092000 | ns | 0.056 | 0.552000 | ns |
| target.fixation.proportion | 1 | 50 | 4.1 | 0.048000 | * | 0.076 | 0.336000 | ns |

**Target Colour × Room × Distractor**

| Effect | DFn | DFd | F | p | <.05 | pes | p_holm | <.05 |
| --- | --- | --- | --- | --- | --- | --- | --- | --- |
| initiation.time | 1 | 53 | 0.877 | 0.353000 | ns | 0.016 | 1.000000 | ns |
| movement.time | 1 | 53 | 1.93 | 0.171000 | ns | 0.035 | 1.000000 | ns |
| response.time | 1 | 53 | 0.26 | 0.612000 | ns | 0.005 | 1.000000 | ns |
| curvature | 1 | 53 | 0.995 | 0.323000 | ns | 0.018 | 1.000000 | ns |
| target.attraction | 1 | 53 | 1.268 | 0.265000 | ns | 0.023 | 1.000000 | ns |
| first.target.fixation | 1 | 50 | 0.374 | 0.543000 | ns | 0.007 | 1.000000 | ns |
| gaze.distance | 1 | 50 | 1.815 | 0.184000 | ns | 0.035 | 1.000000 | ns |
| target.fixation.proportion | 1 | 50 | 0.183 | 0.671000 | ns | 0.004 | 1.000000 | ns |

### Target Location

**Target Location × Room**

| Effect | DFn | DFd | F | p | <.05 | pes | ε | p_holm | <.05 |
| --- | --- | --- | --- | --- | --- | --- | --- | --- | --- |
| initiation.time | 2 | 106 | 0.737 | 0.481000 | ns | 0.014 | 0.954 | 1.000000 | ns |
| movement.time | 2 | 106 | 1.787 | 0.173000 | ns | 0.033 | 0.994 | 1.000000 | ns |
| response.time | 2 | 106 | 1.243 | 0.293000 | ns | 0.023 | 0.994 | 1.000000 | ns |
| curvature | 1.7 | 90.02 | 0.814 | 0.429000 | ns | 0.015 | 0.849 | 1.000000 | ns |
| target.attraction | 2 | 106 | 0.183 | 0.833000 | ns | 0.003 | 0.931 | 1.000000 | ns |
| first.target.fixation | 2 | 100 | 0.721 | 0.489000 | ns | 0.014 | 0.917 | 1.000000 | ns |
| gaze.distance | 2 | 100 | 2.001 | 0.141000 | ns | 0.038 | 0.992 | 1.000000 | ns |
| target.fixation.proportion | 1.78 | 88.85 | 0.113 | 0.871000 | ns | 0.002 | 0.889 | 1.000000 | ns |

**Target Location** **× Distractor**

| Effect | DFn | DFd | F | p | <.05 | pes | ε | p_holm | <.05 |
| --- | --- | --- | --- | --- | --- | --- | --- | --- | --- |
| initiation.time | 2 | 106 | 2.797 | 0.066000 | ns | 0.050 | 0.931 | 0.198000 | ns |
| movement.time | 2 | 106 | 4.328 | 0.016000 | * | 0.075 | 0.990 | 0.070000 | ns |
| response.time | 2 | 106 | 9.9 | 0.000114 | *** | 0.157 | 0.953 | 0.000912 | *** |
| curvature | 2 | 106 | 4.413 | 0.014000 | * | 0.077 | 0.955 | 0.070000 | ns |
| target.attraction | 2 | 106 | 5.179 | 0.007000 | ** | 0.089 | 0.954 | 0.049000 | * |
| first.target.fixation | 2 | 100 | 1.801 | 0.170000 | ns | 0.035 | 0.999 | 0.252000 | ns |
| gaze.distance | 2 | 100 | 5.105 | 0.008000 | ** | 0.093 | 0.979 | 0.049000 | * |
| target.fixation.proportion | 2 | 100 | 2.113 | 0.126000 | ns | 0.041 | 0.969 | 0.252000 | ns |

Response Time – Target Location × Distractor

| Distractor comparison by Target Location: Paired *t* tests  *alternative hypothesis: true mean difference is not equal to 0* | | | | | | | | | | |
| --- | --- | --- | --- | --- | --- | --- | --- | --- | --- | --- |
| target_location | group1 | | group2 | DF | statistic | p | p<.05 | p_holm | <.05 | |
| left | Absent | | Present | 53 | -4.499020 | 0.00003763 | *** | 0.00007526 | *** | |
| top | Absent | | Present | 53 | -9.130232 | 0.00000000 | *** | 0.00000000 | *** | |
| right | Absent | | Present | 53 | -3.607398 | 0.00068494 | *** | 0.00068494 | *** | |
| Target Location comparison: Paired *t* tests  *Comparison of (Distractor Absent - Distractor Present) Difference*  *alternative hypothesis: true mean difference is not equal to 0* | | | | | | | | | | |
| group1 | | group2 | | DF | statistic | p | p<.05 | p_holm | | <.05 |
| left | | top | | 53 | 3.2600048 | 0.00194947 | ** | 0.00389894 | | ** |
| left | | right | | 53 | -0.7670282 | 0.44646821 | ns | 0.44646821 | | ns |
| top | | right | | 53 | -3.9698893 | 0.00021782 | *** | 0.00065346 | | *** |

Target Attraction – Target Location × Distractor

| Distractor comparison by Target Location: Paired *t* tests  *alternative hypothesis: true mean difference is not equal to 0* | | | | | | | | | | |
| --- | --- | --- | --- | --- | --- | --- | --- | --- | --- | --- |
| target_location | group1 | | group2 | DF | statistic | p | p<.05 | p_holm | <.05 | |
| left | Absent | | Present | 53 | 4.077528 | 0.00015354 | *** | 0.00015354 | *** | |
| top | Absent | | Present | 53 | 7.482175 | 0.00000000 | *** | 0.00000000 | *** | |
| right | Absent | | Present | 53 | 4.416837 | 0.00004972 | *** | 0.00009944 | *** | |
| Target Location comparison: Paired *t* tests  *Comparison of (Distractor Absent - Distractor Present) Difference*  *alternative hypothesis: true mean difference is not equal to 0* | | | | | | | | | | |
| group1 | | group2 | | DF | statistic | p | p<.05 | p_holm | | <.05 |
| left | | top | | 53 | -3.2278826 | 0.00214139 | ** | 0.00642417 | | ** |
| left | | right | | 53 | -0.3443042 | 0.73197996 | ns | 0.73197996 | | ns |
| top | | right | | 53 | 2.3205077 | 0.02419398 | * | 0.04838796 | | * |

Gaze Distance – Target Location × Distractor

| Distractor comparison by Target Location: Paired *t* tests  *alternative hypothesis: true mean difference is not equal to 0* | | | | | | | | | | |
| --- | --- | --- | --- | --- | --- | --- | --- | --- | --- | --- |
| target_location | group1 | | group2 | DF | statistic | p | p<.05 | p_holm | <.05 | |
| left | Absent | | Present | 50 | -2.732795 | 0.00865841 | ** | 0.01731682 | * | |
| top | Absent | | Present | 50 | -5.566191 | 0.00000103 | *** | 0.00000309 | *** | |
| right | Absent | | Present | 50 | -0.826057 | 0.41269618 | ns | 0.41269618 | ns | |
| Target Location comparison: Paired *t* tests  *Comparison of (Distractor Absent - Distractor Present) Difference*  *alternative hypothesis: true mean difference is not equal to 0* | | | | | | | | | | |
| group1 | | group2 | | DF | statistic | p | p<.05 | p_holm | | <.05 |
| left | | top | | 50 | 1.967909 | 0.05463699 | ns | 0.10927398 | | ns |
| left | | right | | 50 | -1.135107 | 0.26174289 | ns | 0.26174289 | | ns |
| top | | right | | 50 | -2.811340 | 0.00702821 | ** | 0.02108463 | | * |

**Target Location** **× Room × Distractor**

| Effect | DFn | DFd | F | p | <.05 | pes | ε | p_holm | <.05 |
| --- | --- | --- | --- | --- | --- | --- | --- | --- | --- |
| initiation.time | 2 | 106 | 1.141 | 0.323000 | ns | 0.021 | 0.953 | 1.000000 | ns |
| movement.time | 2 | 106 | 0.311 | 0.733000 | ns | 0.006 | 0.963 | 1.000000 | ns |
| response.time | 2 | 106 | 0.532 | 0.589000 | ns | 0.010 | 0.913 | 1.000000 | ns |
| curvature | 2 | 106 | 3.368 | 0.038000 | * | 0.060 | 0.909 | 0.304000 | ns |
| target.attraction | 2 | 106 | 1.177 | 0.312000 | ns | 0.022 | 0.920 | 1.000000 | ns |
| first.target.fixation | 2 | 100 | 0.489 | 0.615000 | ns | 0.010 | 0.919 | 1.000000 | ns |
| gaze.distance | 1.75 | 87.66 | 1.467 | 0.237000 | ns | 0.029 | 0.877 | 1.000000 | ns |
| target.fixation.proportion | 2 | 100 | 1.25 | 0.291000 | ns | 0.024 | 0.942 | 1.000000 | ns |

## Section 2: Main Effects

### Distractor Presence

Our primary analyses consisted of a series of repeated-measure ANOVAs featuring room (rich, sparse) and distractor presence (present, absent) as within-subject factors for all measures aside from DA. DA was calculated as a difference score reflecting performance on distractor-absent and distractor-present trials and was therefore analyzed using (1) one-sample *t*-tests to determine whether average DA scores differed significantly from zero and (2) paired-samples *t*-tests to determine whether DA scores differed significantly between the rich and sparse virtual environments. To control family-wise error rate, Holm-Bonferroni corrections were applied separately within each effect type, treating the 9 dependent variables as a family for the effects of distractor type (or the distractor effect for DA) and the interaction of distractor and room (or the comparison of distractor effects between rooms for DA), and the 8 dependent variables (not including DA) as a family for the effect of room (α = .05 per family).

| **Distractor Presence** | | | | | | | | |
| --- | --- | --- | --- | --- | --- | --- | --- | --- |
| Effect | DFn | DFd | F | p | <.05 | pes | p_holm | <.05 |
| initiation.time | 1 | 53 | 18.122 | 0.000085 | *** | 0.255 | 0.000170 | *** |
| movement.time | 1 | 53 | 52.18 | 0.000000 | *** | 0.496 | 0.000000 | *** |
| response.time | 1 | 53 | 87.656 | 0.000000 | *** | 0.623 | 0.000000 | *** |
| curvature | 1 | 53 | 50.242 | 0.000000 | *** | 0.487 | 0.000000 | *** |
| target.attraction | 1 | 53 | 75.574 | 0.000000 | *** | 0.588 | 0.000000 | *** |
| first.target.fixation | 1 | 50 | 27.859 | 0.000003 | *** | 0.358 | 0.000011 | *** |
| gaze.distance | 1 | 50 | 24.267 | 0.000010 | *** | 0.327 | 0.000029 | *** |
| target.fixation.proportion | 1 | 50 | 2.703 | 0.106000 | ns | 0.051 | 0.106000 | ns |

Distractor Attraction: One-Sample *t* test

*alternative hypothesis: true mean is not equal to 0*

| Effect | DF | T.Stat | p | <.05 | p_holm | <.05 |
| --- | --- | --- | --- | --- | --- | --- |
| distractor.attraction | 53 | 8.9388 | 3.655e-12 | *** | 2.924e-11 | *** |

| **Room Type** | | | | | | | | |
| --- | --- | --- | --- | --- | --- | --- | --- | --- |
| Effect | DFn | DFd | F | p | <.05 | pes | p_holm | <.05 |
| initiation.time | 1 | 53 | 13.343 | 0.000595 | *** | 0.201 | 0.002975 | ** |
| movement.time | 1 | 53 | 2.244 | 0.140000 | ns | 0.041 | 0.560000 | ns |
| response.time | 1 | 53 | 16.431 | 0.000166 | *** | 0.237 | 0.001162 | ** |
| curvature | 1 | 53 | 0.047 | 0.829000 | ns | 0.001 | 1.000000 | ns |
| target.attraction | 1 | 53 | 0.000881 | 0.976000 | ns | 0.000 | 1.000000 | ns |
| first.target.fixation | 1 | 50 | 1.015 | 0.319000 | ns | 0.020 | 0.957000 | ns |
| gaze.distance | 1 | 50 | 18.745 | 0.000072 | *** | 0.273 | 0.000574 | *** |
| target.fixation.proportion | 1 | 50 | 16.219 | 0.000192 | *** | 0.245 | 0.001162 | ** |

| **Distractor Presence × Room Type** | | | | | | | | |
| --- | --- | --- | --- | --- | --- | --- | --- | --- |
| Effect | DFn | DFd | F | p | <.05 | pes | p_holm | <.05 |
| initiation.time | 1 | 53 | 0.667 | 0.418000 | ns | 0.012 | 0.927000 | ns |
| movement.time | 1 | 53 | 3.25 | 0.077000 | ns | 0.058 | 0.616000 | ns |
| response.time | 1 | 53 | 0.447 | 0.506000 | ns | 0.008 | 0.927000 | ns |
| curvature | 1 | 53 | 2.552 | 0.116000 | ns | 0.046 | 0.616000 | ns |
| target.attraction | 1 | 53 | 1.77 | 0.189000 | ns | 0.032 | 0.756000 | ns |
| first.target.fixation | 1 | 50 | 2.988 | 0.090000 | ns | 0.056 | 0.616000 | ns |
| gaze.distance | 1 | 50 | 3.12 | 0.083000 | ns | 0.059 | 0.616000 | ns |
| target.fixation.proportion | 1 | 50 | 1.056 | 0.309000 | ns | 0.021 | 0.927000 | ns |

Distractor Attraction: Paired *t* test

*alternative hypothesis: true mean difference is not equal to 0*

| group1 | group2 | DF | statistic | p | <.05 | p_holm | <.05 |
| --- | --- | --- | --- | --- | --- | --- | --- |
| Rich | Blank | 53 | -0.9460 | 0.3484 | ns | 1.000 | ns |

Bayes Factors for Distractor Presence × Room Type (computed using <https://tomfaulkenberry.shinyapps.io/psystat>)

IT: BF_01_= 4.19

MT: BF_01_= 1.29

RT: BF_01_= 4.64

CURV: BF_01_= 1.77

TA: BF_01_= 2.52

FTF: BF_01_= 1.42

GD: BF_01_= 1.34

TFP: BF_01_= 3.40

## Section 3: Exploratory Analysis of Distractor Type

### Distractor Type Differences

We conducted a series of repeated-measure ANOVAs featuring room (rich, sparse) and distractor type (Near.Side, Near.Top, Far) as within-subject factors. Note that the None distractor type was not featured in these ANOVAs because each of the measures were calculated using a difference score that featured the distractor absent trials. To control family-wise error rate, Holm-Bonferroni corrections were applied separately within each effect (distractor type, room type, distractor type × room type), treating the 9 dependent variables as a family (α = .05 per family). If a main effect of distractor type was not observed in an ANOVA, we conducted a one-sample *t*-test comparing the average score across all three distractor types to zero. If a main effect of distractor type was observed, paired-samples *t*-tests were performed, with Holm-Bonferroni corrections applied to account for the three comparisons performed. All reported *p*-values in the written summary below are adjusted to reflect the Holm-Bonferroni corrections. Greenhouse-Geisser corrections were applied to all tests that violated the assumption of sphericity. Any main effects not explicitly reported failed to reach significance.

***Temporal Measures.*** IT differences revealed a main effect of distractor type, *F*(1.54, 81.84) = 12.94, ε = .77, *p* < .001, η_p_^2^ = .20, with larger distractor effects on Near.Side trials (*M* = 27.8ms, *SD* = 47.7ms) than Far trials (*M* = -8.4ms, *SD* = 40.4ms), *t*(53) = 3.92, *p* < .001, and larger distractor effects on Near.Top trials (*M* = 29.4ms, *SD* = 45.1ms) than Far trials, *t*(53) = 6.78, *p* < .001, but no significant difference between Near.Side trials and Near.Top trials, *t*(53) = -0.17, *p* = .87. Follow-up *t*-tests confirmed the presence of a significant distractor effect on Near.Side trials, *t*(53) = 4.28, *p* < .001, and Near.Top trials, *t*(53) = 4.79, *p* < .001, but not Far trials, *t*(53) = -1.53, *p* = .133.

MT differences revealed a main effect of distractor type, *F*(1.67, 88.26) = 12.22, ε = .83, *p* < .001, η_p_^2^ = .19, with larger distractor effects on Near.Side trials (*M* = 47.5ms, *SD* = 51.6ms) than Far trials (*M* = 11.3ms, *SD* = 37.5ms), *t*(53) = 4.29, *p* < .001, and larger distractor effects on Near.Top trials (*M* = 35.2ms, *SD* = 45.2ms) than Far trials, *t*(53) = 4.27, *p* < .001, but no significant difference between Near.Side trials and Near.Top trials, *t*(53) = 1.56, *p* = .12. Follow-up *t*-tests confirmed the presence of a significant distractor effect on Near.Side trials, *t*(53) = 6.77, *p* < .001, Near.Top trials, *t*(53) = 5.86, *p* < .001, and Far trials, *t*(53) = 2.21, *p* = .032.

RT differences revealed a main effect of distractor type, *F*(1.59, 84.23) = 30.87, ε = .80, *p* < .001, η_p_^2^ = .37, with larger distractor effects on Near.Side trials (*M* = 75.3ms, *SD* = 59.2ms) than Far trials (*M* = 2.9ms, *SD* = 53.9ms), *t*(53) = 6.47, *p* < .001, and larger distractor effects on Near.Top trials (*M* = 64.6ms, *SD* = 54.8ms) than Far trials, *t*(53) = 8.92, *p* < .001, but no significant difference between Near.Side trials and Near.Top trials, *t*(53) = 0.99, *p* = .32. Follow-up *t*-tests confirmed the presence of a significant distractor effect on Near.Side trials, *t*(53) = 9.35, *p* < .001, and Near.Top trials, *t*(53) = 8.66, *p* < .001, but not Far trials, *t*(53) = 0.39, *p* = .70.

***Reach Measures.*** CURV differences revealed a significant main effect of distractor type, *F*(2, 106), = 21.57, *p* < .001, η_p_^2^ = .29, with larger distractor effects on Near.Side trials (*M* = 0.034, *SD* = 0.045) than Far trials (*M* = -0.003, *SD* = 0.024), *t*(53) = 5.00, *p* < .001, and larger distractor effects on Near.Top trials (*M* = 0.037, *SD* = 0.039) than Far trials, *t*(53) = 6.59, *p* < .001, but no significant difference between Near.Side trials and Near.Top trials, *t*(53) = -0.48, *p* = .63. Follow-up *t*-tests confirmed the presence of a significant distractor effect on Near.Side trials, *t*(53) = 5.58, *p* < .001, and Near.Top trials, *t*(53) = 7.04, *p* < .001, but not Far trials, *t*(53) = -0.79, *p* = .44.

TA differences revealed a significant main effect of distractor type, *F*(1.79, 94.92), = 16.47, ε = .896, *p* < .001, η_p_^2^ = .24, with larger distractor effects on Near.Side trials (*M* = -4.00%, *SD* = 3.91%) than Far trials (*M* = -0.76%, *SD* = 2.74%), *t*(53) = -4.77, *p* < .001, and larger distractor effects on Near.Top trials (*M* = -3.44%, *SD* = 3.55%) than Far trials, *t*(53) = -5.28, *p* < .001, but no significant difference between Near.Side trials and Near.Top trials, *t*(53) = -0.94, *p* = .35. Follow-up *t*-tests confirmed a significant distractor effect on Near.Side trials, *t*(53) = -7.53, *p* < .001, Near.Top trials, *t*(53) = -7.13, *p* < .001, and Far trials, *t*(53) = -2.03, *p* = .047.

Finally, DA differences revealed a significant main effect of distractor type, *F*(2, 106), = 5.20, *p* = .021, η_p_^2^ = .09, with significantly more attraction to the distractor on Near.Top trials (*M* = 2.60%, *SD* = 2.68%) than Far trials (*M =* 1.38%, *SD* = 1.74%), *t*(53) = 3.29, *p* = .005, but no significant differences between Near.Side trials (*M* = 1.78%, *SD* = 2.28%) and Far trials, *t*(53) = 1.01, *p* = .32, or between Near.Side trials and Near.Top trials, *t*(53) = -2.13, *p* = .076. Follow-up *t*-tests revealed significant distractor effects for each distractor type, with significant attraction to the distractor on Near.Side trials, *t*(53) = 5.72, *p* < .001, Near.Top trials, *t*(53) = 7.12, *p* < .001, and Far trials, *t*(53) = 5.83, *p* < .001.

***Gaze Measures.*** FTF differences did not reveal a main effect of distractor type, *F*(2, 100), = 1.34, *p* = .798, η_p_^2^ = .03, with comparable differences for Near.Side trials (*M =* 33.9ms, *SD* = 68.2ms), Near.Top trials (*M* = 23.5ms, *SD* = 53.5ms), and Far trials (*M =* 17.1ms, *SD* = 51.3ms). A follow-up one-sample *t*-test revealed a significant distractor effect for average FTF difference scores, *t*(50) = 4.73, *p* < .001.

GD differences revealed a main effect of distractor type, *F*(2, 100), = 17.24, *p* < .001, η_p_^2^ = .26, with larger distractor effects on Near.Side trials (*M =* 0.110rad, *SD* = 0.141rad) than Far trials (*M =* -0.032rad, *SD* = 0.150rad), *t*(50) = 4.77, *p* < .001, and larger distractor effects on Near.Top trials (*M* = 0.108rad, *SD* = 0.132rad) compared to Far trials, *t*(50) = 5.79, *p* < .001, but no significant difference between Near.Side trials and Near.Top trials, *t*(50) = 0.09, *p* = .92. Follow-up *t*-tests revealed significant distractor effects on Near.Side trials, *t*(50) = 5.58, *p* < .001, and Near.Top trials, *t*(50) = 5.82, *p* < .001, but not Far trials, *t*(50) = -1.54, *p* = .13.

TFP differences revealed no main effect of distractor type, *F*(1.48, 73.86), = 3.30, ε = .74, *p* = .168, η_p_^2^ = .06, with comparable distractor effects on Near.Side trials (*M =* -0.85%, *SD* = 2.45%), Near.Top trials, (*M* = -0.02%, *SD* = 2.22%), and Far trials (*M =* 0.02%, *SD* = 2.31%). A follow-up one-sample *t*-test showed no significant effect for average TFP difference scores, *t*(50) = -1.23, *p* = .22.

Bayes Factors for the paired-samples *t*-tests comparing Near.Side and Near.Top trials (computed using <https://tomfaulkenberry.shinyapps.io/psystat>):

IT: BF_01_ = 6.65

MT: BF_01_ = 2.16

RT: BF_01_ = 4.24

CURV: BF_01_ = 6.04

TA: BF_01_ = 4.43

DA: BF_10_ = 1.19

GD: BF_01_ = 6.54

| **Distractor Type** | | | | | | | | | |
| --- | --- | --- | --- | --- | --- | --- | --- | --- | --- |
| Effect | DFn | DFd | F | p | <.05 | pes | ε | p_holm | <.05 |
| initiation.time.diff | 1.54 | 81.84 | 12.937 | 0.000067 | *** | 0.196 | 0.772 | 0.000324 | *** |
| movement.time.diff | 1.67 | 88.26 | 12.216 | 0.000065 | *** | 0.187 | 0.833 | 0.000324 | *** |
| response.time.diff | 1.59 | 84.23 | 30.873 | 0.000000 | *** | 0.368 | 0.795 | 0.000000 | *** |
| curvature.diff | 2 | 106 | 21.568 | 0.000000 | *** | 0.289 | 0.947 | 0.000000 | *** |
| target.attraction.diff | 1.79 | 94.92 | 16.473 | 0.000002 | *** | 0.237 | 0.896 | 0.000011 | *** |
| distractor.attraction.diff | 2 | 106 | 5.202 | 0.007000 | ** | 0.089 | 0.994 | 0.021000 | * |
| first.target.fixation.diff | 2 | 100 | 1.34 | 0.266000 | ns | 0.026 | 0.941 | 0.266000 | ns |
| gaze.distance.diff | 2 | 100 | 17.239 | 0.000000 | *** | 0.256 | 0.951 | 0.000003 | *** |
| target.fixation.proportion.diff | 1.48 | 73.86 | 3.304 | 0.056000 | ns | 0.062 | 0.739 | 0.112000 | ns |

| **Room Type** | | | | | | | | |
| --- | --- | --- | --- | --- | --- | --- | --- | --- |
| Effect | DFn | DFd | F | p | <.05 | pes | p_holm | <.05 |
| initiation.time.diff | 1 | 53 | 0.678 | 0.414000 | ns | 0.013 | 1 | ns |
| movement.time.diff | 1 | 53 | 3.103 | 0.084000 | ns | 0.055 | 0.702 | ns |
| response.time.diff | 1 | 53 | 0.434 | 0.513000 | ns | 0.008 | 1 | ns |
| curvature.diff | 1 | 53 | 2.684 | 0.107000 | ns | 0.048 | 0.749 | ns |
| target.attraction.diff | 1 | 53 | 2.067 | 0.156000 | ns | 0.038 | 0.936 | ns |
| distractor.attraction.diff | 1 | 53 | 0.912 | 0.344000 | ns | 0.017 | 1 | ns |
| first.target.fixation.diff | 1 | 50 | 1.056 | 0.309000 | ns | 0.021 | 1 | ns |
| gaze.distance.diff | 1 | 50 | 3.24 | 0.078000 | ns | 0.061 | 0.702 | ns |
| target.fixation.proportion.diff | 1 | 50 | 0.584 | 0.448000 | ns | 0.012 | 1 | ns |

Bayes Factors for Room Type (computed using <https://tomfaulkenberry.shinyapps.io/psystat>)

IT: BF_01_= 4.17

MT: BF_01_= 1.38

RT: BF_01_= 4.67

CURV: BF_01_= 1.67

TA: BF_01_= 2.20

DA: BF_01_= 3.74

FTF: BF_01_= 3.40

GD: BF_01_= 1.27

TFP: BF_01_= 4.23

| **Distractor Type × Room Type** | | | | | | | | | |
| --- | --- | --- | --- | --- | --- | --- | --- | --- | --- |
| Effect | DFn | DFd | F | p | <.05 | pes | ε | p_holm | <.05 |
| initiation.time.diff | 2 | 106 | 0.635 | 0.532000 | ns | 0.012 | 0.906 | 1.000000 | ns |
| movement.time.diff | 1.79 | 94.91 | 0.536 | 0.567000 | ns | 0.010 | 0.895 | 1.000000 | ns |
| response.time.diff | 1.67 | 88.33 | 0.336 | 0.676000 | ns | 0.006 | 0.833 | 1.000000 | ns |
| curvature.diff | 2 | 106 | 2.608 | 0.078000 | ns | 0.047 | 0.928 | 0.702000 | ns |
| target.attraction.diff | 2 | 106 | 1.005 | 0.369000 | ns | 0.019 | 0.947 | 1.000000 | ns |
| distractor.attraction.diff | 2 | 106 | 0.032 | 0.968000 | ns | 0.001 | 0.968 | 1.000000 | ns |
| first.target.fixation.diff | 1.6 | 79.85 | 1.844 | 0.172000 | ns | 0.036 | 0.799 | 1.000000 | ns |
| gaze.distance.diff | 1.58 | 79.23 | 0.97 | 0.366000 | ns | 0.019 | 0.792 | 1.000000 | ns |
| target.fixation.proportion.diff | 1.55 | 77.25 | 2.02 | 0.150000 | ns | 0.039 | 0.773 | 1.000000 | ns |

Bayes Factors for Distractor Type × Room Type (computed using <https://tomfaulkenberry.shinyapps.io/psystat>)

IT: BF_01_= 28.43

MT: BF_01_= 20.54

RT: BF_01_= 18.96

CURV: BF_01_= 4.42

TA: BF_01_= 19.95

DA: BF_01_= 50.89

FTF: BF_01_= 5.02

GD: BF_01_= 9.38

TFP: BF_01_= 4.15

**Initiation Time Difference**

One sample *t* tests

*alternative hypothesis: true mean is not equal to 0*

| Distractor | DF | T.Stat | p | <.05 | p_holm | <.05 |
| --- | --- | --- | --- | --- | --- | --- |
| Near.Side | 53 | 4.282755 | 0.000078 | *** | 0.000156 | *** |
| Near.Top | 53 | 4.787467 | 0.000014 | *** | 0.000042 | *** |
| Far | 53 | -1.527095 | 0.132684 | ns | 0.132684 | ns |

Paired *t* tests

*alternative hypothesis: true mean difference is not equal to 0*

| Group1 | Group2 | DF | T.Stat | p | <.05 | p_holm | <.05 |
| --- | --- | --- | --- | --- | --- | --- | --- |
| Near.Side | Near.Top | 53 | -0.1677167 | 0.867445 | ns | 0.867445 | ns |
| Near.Side | Far | 53 | 3.9200157 | 0.000256 | *** | 0.000512 | *** |
| Near.Top | Far | 53 | 6.7750208 | 0.000000 | *** | 0.000000 | *** |

**Movement Time Difference**

One sample *t* tests

*alternative hypothesis: true mean is not equal to 0*

| Distractor | DF | T.Stat | p | <.05 | p_holm | <.05 |
| --- | --- | --- | --- | --- | --- | --- |
| Near.Side | 53 | 6.771388 | 0.000000 | *** | 0.000000 | *** |
| Near.Top | 53 | 5.858532 | 0.000000 | *** | 0.000001 | *** |
| Far | 53 | 2.208112 | 0.031585 | * | 0.031585 | * |

Paired *t* tests

*alternative hypothesis: true mean difference is not equal to 0*

| Group1 | Group2 | DF | T.Stat | p | <.05 | p_holm | <.05 |
| --- | --- | --- | --- | --- | --- | --- | --- |
| Near.Side | Near.Top | 53 | 1.563955 | 0.123780 | ns | 0.123780 | ns |
| Near.Side | Far | 53 | 4.289118 | 0.000076 | *** | 0.000229 | *** |
| Near.Top | Far | 53 | 4.267678 | 0.000082 | *** | 0.000229 | *** |

**Response Time Difference**

One sample *t* tests

*alternative hypothesis: true mean is not equal to 0*

| Distractor | DF | T.Stat | p | <.05 | p_holm | <.05 |
| --- | --- | --- | --- | --- | --- | --- |
| Near.Side | 53 | 9.3530249 | 0.000000 | *** | 0.000000 | *** |
| Near.Top | 53 | 8.6606574 | 0.000000 | *** | 0.000000 | *** |
| Far | 53 | 0.3910396 | 0.697336 | ns | 0.697336 | ns |

Paired *t* tests

*alternative hypothesis: true mean difference is not equal to 0*

| Group1 | Group2 | DF | T.Stat | p | <.05 | p_holm | <.05 |
| --- | --- | --- | --- | --- | --- | --- | --- |
| Near.Side | Near.Top | 53 | 0.9871917 | 0.328034 | ns | 0.328034 | ns |
| Near.Side | Far | 53 | 6.4710027 | 0.000000 | *** | 0.000000 | *** |
| Near.Top | Far | 53 | 8.9249457 | 0.000000 | *** | 0.000000 | *** |

**Curvature Difference**

One sample *t* tests

*alternative hypothesis: true mean is not equal to 0*

| Distractor | DF | T.Stat | p | <.05 | p_holm | <.05 |
| --- | --- | --- | --- | --- | --- | --- |
| Near.Side | 53 | 5.5779756 | 0.000001 | *** | 0.000002 | *** |
| Near.Top | 53 | 7.0418094 | 0.000000 | *** | 0.000000 | *** |
| Far | 53 | -0.7853541 | 0.435743 | ns | 0.435743 | ns |

Paired *t* tests

*alternative hypothesis: true mean difference is not equal to 0*

| Group1 | Group2 | DF | T.Stat | p | <.05 | p_holm | <.05 |
| --- | --- | --- | --- | --- | --- | --- | --- |
| Near.Side | Near.Top | 53 | -0.4821125 | 0.631711 | ns | 0.631711 | ns |
| Near.Side | Far | 53 | 4.9988262 | 0.000007 | *** | 0.000013 | *** |
| Near.Top | Far | 53 | 6.5898169 | 0.000000 | *** | 0.000000 | *** |

**Target Attraction Difference**

One sample *t* tests

*alternative hypothesis: true mean is not equal to 0*

| Distractor | DF | T.Stat | p | <.05 | p_holm | <.05 |
| --- | --- | --- | --- | --- | --- | --- |
| Near.Side | 53 | -7.526238 | 0.000000 | *** | 0.000000 | *** |
| Near.Top | 53 | -7.133468 | 0.000000 | *** | 0.000000 | *** |
| Far | 53 | -2.034525 | 0.046915 | * | 0.046915 | * |

Paired *t* tests

*alternative hypothesis: true mean difference is not equal to 0*

| Group1 | Group2 | DF | T.Stat | p | <.05 | p_holm | <.05 |
| --- | --- | --- | --- | --- | --- | --- | --- |
| Near.Side | Near.Top | 53 | -0.9410239 | 0.350962 | ns | 0.350962 | ns |
| Near.Side | Far | 53 | -4.7675073 | 0.000015 | *** | 0.000030 | *** |
| Near.Top | Far | 53 | -5.2847757 | 0.000002 | *** | 0.000007 | *** |

**Distractor Attraction Difference**

One sample *t* tests

*alternative hypothesis: true mean is not equal to 0*

| Distractor | DF | T.Stat | p | <.05 | p_holm | <.05 |
| --- | --- | --- | --- | --- | --- | --- |
| Near.Side | 53 | 5.722555 | 0.000000 | *** | 0.000001 | *** |
| Near.Top | 53 | 7.121304 | 0.000000 | *** | 0.000000 | *** |
| Far | 53 | 5.833647 | 0.000000 | *** | 0.000001 | *** |

Paired *t* tests

*alternative hypothesis: true mean difference is not equal to 0*

| Group1 | Group2 | DF | T.Stat | p | <.05 | p_holm | <.05 |
| --- | --- | --- | --- | --- | --- | --- | --- |
| Near.Side | Near.Top | 53 | -2.130196 | 0.037816 | * | 0.075631 | ns |
| Near.Side | Far | 53 | 1.013637 | 0.315361 | ns | 0.315361 | ns |
| Near.Top | Far | 53 | 3.288258 | 0.001794 | ** | 0.005383 | ** |

| **First Target Fixation Difference** | | | | | |
| --- | --- | --- | --- | --- | --- |
| One sample *t* tests  *alternative hypothesis: true mean is not equal to 0* | | | | |  |
| Distractor | DF | T.Stat | p | p<.05 | |
| All | 50 | 4.7296 | 0.000019 | **** | |

**Gaze Distance Difference**

One sample *t* tests

*alternative hypothesis: true mean is not equal to 0*

| Distractor | DF | T.Stat | p | <.05 | p_holm | <.05 |
| --- | --- | --- | --- | --- | --- | --- |
| Near.Side | 50 | 5.578116 | 0.000001 | *** | 0.000002 | *** |
| Near.Top | 50 | 5.818991 | 0.000000 | *** | 0.000001 | *** |
| Far | 50 | -1.544995 | 0.128654 | ns | 0.128654 | ns |

Paired *t* tests

*alternative hypothesis: true mean difference is not equal to 0*

| Group1 | Group2 | DF | T.Stat | p | <.05 | p_holm | <.05 |
| --- | --- | --- | --- | --- | --- | --- | --- |
| Near.Side | Near.Top | 50 | 0.0930699 | 0.926220 | ns | 0.926220 | ns |
| Near.Side | Far | 50 | 4.7746476 | 0.000016 | *** | 0.000032 | *** |
| Near.Top | Far | 50 | 5.7937739 | 0.000000 | *** | 0.000001 | *** |

| **Target Fixation Proportion Difference** | | | | | |
| --- | --- | --- | --- | --- | --- |
| One sample *t* tests  *alternative hypothesis: true mean is not equal to 0* | | | | |  |
| Distractor | DF | T.Stat | p | p<.05 | |
| All | 50 | -1.2337 | 0.2231 | ns | |

## Section 4: Exploratory Correlation Analyses

To assess correlations between performance measures, on each trial we calculated the difference in each measure from the average for that participant. For each participant, pearson's correlations coefficients were calculated between the difference scores for each measure, which were converted to z-values using a Fisher transform. For each pair of measures a one-sample t-test was conducted on the z-values calculated for each participant (with each z-value representing a correlation for one participant). After applying Bonferroni corrections, an alpha level of .05 was adopted. Finally, mean z-values for each pair of measures were transformed into correlation coefficients for reporting.

| Distractor = None | | | | | | | | |
| --- | --- | --- | --- | --- | --- | --- | --- | --- |
|  | init.time.diff | movement.time.diff | response.time.diff | curvature.diff | target.attraction.diff | first.fix.target.diff | gaze.distance.diff |  |
| init.time.diff |  |  |  |  |  |  |  |  |
| movement.time.diff | r = -0.2106 p < .001 |  |  |  |  |  |  |  |
| response.time.diff | r = 0.6653 p < .001 | r = 0.623 p < .001 |  |  |  |  |  |  |
| curvature.diff | r = -0.1377 p < .001 | r = 0.5516 p < .001 | r = 0.3176 p < .001 |  |  |  |  |  |
| target.attraction.diff | r = 0.1829 p < .001 | r = -0.7536 p < .001 | r = -0.4356 p < .001 | r = -0.7455 p < .001 |  |  |  |  |
| first.fix.target.diff | r = 0.0826 p < .001 | r = 0.1042 p < .001 | r = 0.1525 p < .001 | r = 0.1066 p = 0.0026 | r = -0.0565 p = 0.3322 |  |  |  |
| gaze.distance.diff | r = 0.4853 p < .001 | r = 0.4584 p < .001 | r = 0.7538 p < .001 | r = 0.2159 p < .001 | r = -0.3215 p < .001 | r = 0.0001 p = 1 |  |  |
| target.gaze.diff | r = -0.0445 p = 0.6004 | r = -0.0815 p < .001 | r = -0.1091 p < .001 | r = -0.1294 p < .001 | r = 0.0745 p < .001 | r = -0.5986 p < .001 | r = -0.1837 p < .001 |  |
| Distractor = Near.Side | | | | | | | | |
| Near.Side | init.time.diff | movement.time.diff | response.time.diff | curvature.diff | target.attraction.diff | distractor.attraction.diff | first.fix.target.diff | gaze.distance.diff |
| init.time.diff |  |  |  |  |  |  |  |  |
| movement.time.diff | r = -0.3185 p < .001 |  |  |  |  |  |  |  |
| response.time.diff | r = 0.6294 p < .001 | r = 0.6066 p < .001 |  |  |  |  |  |  |
| curvature.diff | r = -0.2333 p < .001 | r = 0.6867 p < .001 | r = 0.3642 p < .001 |  |  |  |  |  |
| target.attraction.diff | r = 0.3142 p < .001 | r = -0.8219 p < .001 | r = -0.4211 p < .001 | r = -0.8362 p < .001 |  |  |  |  |
| distractor.attraction.diff | r = -0.2317 p < .001 | r = 0.6039 p < .001 | r = 0.2997 p < .001 | r = 0.6846 p < .001 | r = -0.7945 p < .001 |  |  |  |
| first.fix.target.diff | r = 0.0407 p = 1 | r = 0.0355 p = 1 | r = 0.0868 p = 0.4698 | r = -0.0023 p = 1 | r = -0.0011 p = 1 | r = 0.0153 p = 1 |  |  |
| gaze.distance.diff | r = 0.4529 p < .001 | r = 0.4358 p < .001 | r = 0.7556 p < .001 | r = 0.2562 p < .001 | r = -0.3112 p < .001 | r = 0.2076 p < .001 | r = -0.0525 p = 1 |  |
| target.gaze.diff | r = -0.0361 p = 1 | r = -0.1075 p = 0.0073 | r = -0.1446 p = 0.0104 | r = -0.0747 p = 0.1454 | r = 0.0837 p = 0.0499 | r = -0.0784 p = 0.0436 | r = -0.6298 p < .001 | r = -0.2081 p = 0.0038 |
| Distractor = Near.Top | | | | | | | | |
| Near.Side | init.time.diff | movement.time.diff | response.time.diff | curvature.diff | target.attraction.diff | distractor.attraction.diff | first.fix.target.diff | gaze.distance.diff |
| init.time.diff |  |  |  |  |  |  |  |  |
| movement.time.diff | r = -0.1604 p < .001 |  |  |  |  |  |  |  |
| response.time.diff | r = 0.6649 p < .001 | r = 0.6876 p < .001 |  |  |  |  |  |  |
| curvature.diff | r = -0.1137 p = 0.0012 | r = 0.6519 p < .001 | r = 0.4164 p < .001 |  |  |  |  |  |
| target.attraction.diff | r = 0.1466 p < .001 | r = -0.787 p < .001 | r = -0.5111 p < .001 | r = -0.8151 p < .001 |  |  |  |  |
| distractor.attraction.diff | r = -0.0863 p = 0.2367 | r = 0.6171 p < .001 | r = 0.3986 p < .001 | r = 0.8375 p < .001 | r = -0.9019 p < .001 |  |  |  |
| first.fix.target.diff | r = 0.1799 p < .001 | r = 0.2225 p < .001 | r = 0.3292 p < .001 | r = 0.1034 p = 0.3661 | r = -0.1552 p = 0.0029 | r = 0.1127 p = 0.0548 |  |  |
| gaze.distance.diff | r = 0.4877 p < .001 | r = 0.4987 p < .001 | r = 0.7609 p < .001 | r = 0.294 p < .001 | r = -0.3742 p < .001 | r = 0.3009 p < .001 | r = 0.1097 p = 0.3056 |  |
| target.gaze.diff | r = -0.0842 p = 0.0565 | r = -0.1322 p < .001 | r = -0.1749 p < .001 | r = -0.0676 p = 0.2577 | r = 0.1151 p < .001 | r = -0.0816 p = 0.0467 | r = -0.4776 p < .001 | r = -0.2515 p < .001 |
| Distractor = Far | | | | | | | | |
| Near.Side | init.time.diff | movement.time.diff | response.time.diff | curvature.diff | target.attraction.diff | distractor.attraction.diff | first.fix.target.diff | gaze.distance.diff |
| init.time.diff | r = 1 p < .001 |  |  |  |  |  |  |  |
| movement.time.diff | r = -0.1558 p < .001 |  |  |  |  |  |  |  |
| response.time.diff | r = 0.6804 p < .001 | r = 0.6763 p < .001 |  |  |  |  |  |  |
| curvature.diff | r = -0.0663 p = 0.5203 | r = 0.5646 p < .001 | r = 0.3814 p < .001 |  |  |  |  |  |
| target.attraction.diff | r = 0.1312 p = 0.0013 | r = -0.7778 p < .001 | r = -0.5043 p < .001 | r = -0.7113 p < .001 |  |  |  |  |
| distractor.attraction.diff | r = -0.1579 p < .001 | r = 0.6455 p < .001 | r = 0.3689 p < .001 | r = 0.4099 p < .001 | r = -0.8345 p < .001 |  |  |  |
| first.fix.target.diff | r = 0.2079 p < .001 | r = 0.2046 p < .001 | r = 0.3326 p < .001 | r = 0.103 p = 0.1326 | r = -0.1474 p = 0.01 | r = 0.1294 p = 0.0241 |  |  |
| gaze.distance.diff | r = 0.4802 p < .001 | r = 0.501 p < .001 | r = 0.7588 p < .001 | r = 0.2857 p < .001 | r = -0.3756 p < .001 | r = 0.2725 p < .001 | r = 0.1301 p = 0.218 |  |
| target.gaze.diff | r = -0.0596 p = 1 | r = -0.1134 p = 0.0387 | r = -0.1459 p = 0.0039 | r = -0.0812 p = 0.0313 | r = 0.1159 p = 0.0018 | r = -0.0726 p = 0.0953 | r = -0.5051 p < .001 | r = -0.2492 p < .001 |

## Section 5: Two-Dimensional Reach Attraction

In this section we compare reach attraction effects calculated using three dimensions (x, y, z) and using two dimensions (x, y). As in the primary results, repeated-measures ANOVAs feature room (sparse, rich) and distractor presence (present, absent) as within-subject factors for the main distractor effects, and feature room (sparse, rich) and distractor type (Near.Side, Near.Top, Far) as within-subject factors for the exploratory analysis of distractor types. Greenhouse-Geisser corrections were applied to all tests that violated the assumption of sphericity. As the purpose of these results is to compare effects between the two- and three-dimensional calculations, Holm-Bonferroni correction was not applied. Additionally, we report the significant clusters identified in the cluster-based permutation analyses, for each distractor type.

| **Target Attraction** | | | | | | |
| --- | --- | --- | --- | --- | --- | --- |
| 3D | | | | | | |
| Effect | DFn | DFd | F | p | p<.05 | pes |
| distractor | 1 | 53 | 75.574000 | 8.88e-12 | *** | 0.588 |
| room | 1 | 53 | 0.000881 | 9.76e-01 | ns | 0.0000166 |
| distractor:room | 1 | 53 | 1.770000 | 1.89e-01 | ns | 0.032 |
| 2D | | | | | | |
| Effect | DFn | DFd | F | p | p<.05 | pes |
| distractor | 1 | 53 | 72.079 | 1.86e-11 | *** | 0.576 |
| room | 1 | 53 | 0.235 | 6.30e-01 | ns | 0.004 |
| distractor:room | 1 | 53 | 1.941 | 1.69e-01 | ns | 0.035 |

| **Distractor Attraction Difference** | |
| --- | --- |
| 3D | |
| Distractor Presence | Room Type |
| *alternative hypothesis: true mean is not equal to 0* | *alternative hypothesis: true mean difference is not equal to 0* |
| t = 8.9388, df = 53,  p-value = 3.655e-12  95% confidence interval:  1.474223 2.327216  sample estimates: mean of x  1.90072 | t = 0.94601, df = 53,  p-value = 0.3484  95% confidence interval:  -0.3571919 0.9949134  sample estimates: mean difference  0.3188608 |
| 2D | |
| Distractor Presence | Room Type |
| *alternative hypothesis: true mean is not equal to 0* | *alternative hypothesis: true mean difference is not equal to 0* |
| t = 8.7877, df = 53,  p-value = 6.308e-12  95% confidence interval:  1.454135 2.314243  sample estimates: mean of x  1.884189 | t = 0.73249, df = 53,  p-value = 0.4671  95% confidence interval:  -0.4443044 0.9555065  sample estimates: mean difference  0.255601 |

| **Target Attraction Difference** | | | | | | | | | | | | | | | |
| --- | --- | --- | --- | --- | --- | --- | --- | --- | --- | --- | --- | --- | --- | --- | --- |
| 3D - ANOVA | | | | | | | | | | | | | | | |
| Effect | | | DFn | DFd | | | F | | | p | | p<.05 | | | pes |
| distractor_type | | | 1.79 | 94.92 | | | 16.473 | | | 1.90e-06 | | *** | | | 0.237 |
| room | | | 1 | 53 | | | 2.067 | | | 1.56e-01 | | ns | | | 0.038 |
| distractor_type:room | | | 2 | 106 | | | 1.005 | | | 3.69e-01 | | ns | | | 0.019 |
| 2D - ANOVA | | | | | | | | | | | | | | | |
| Effect | | | DFn | DFd | | | F | | | p | | p<.05 | | | pes |
| distractor_type | | | 2 | 106 | | | 14.549 | | | 2.61e-06 | | *** | | | 0.215 |
| room | | | 1 | 53 | | | 2.220 | | | 1.42e-01 | | ns | | | 0.040 |
| distractor_type:room | | | 2 | 106 | | | 0.814 | | | 4.46e-01 | | ns | | | 0.015 |
|  | 3D - One sample *t* tests  *alternative hypothesis: true mean is not equal to 0* | | | | | | | | | | | | | | |
|  | Distractor | | DF | | T.Stat | | | | p | | | | p<.05 | | |
|  | Near.Side | | 53 | | -7.526238 | | | | 6.423088e-10 | | | | *** | | |
|  | Near.Top | | 53 | | -7.133468 | | | | 2.750178e-09 | | | | *** | | |
|  | Far | | 53 | | -2.034525 | | | | 0.046915 | | | | * | | |
|  | 2D - One sample *t* tests  *alternative hypothesis: true mean is not equal to 0* | | | | | | | | | | | | | | |
|  | Distractor | | DF | | T.Stat | | | | p | | | | p<.05 | | |
|  | Near.Side | | 53 | | -7.129807 | | | | 2.787743e-09 | | | | *** | | |
|  | Near.Top | | 53 | | -7.247762 | | | | 1.800683e-09 | | | | *** | | |
|  | Far | | 53 | | -2.270303 | | | | 2.728049e-02 | | | | * | | |
| 3D - Paired *t* tests  *alternative hypothesis: true mean is not equal to 0* | | | | | | | | | | | | | | | |
| Group1 | | Group2 | | | | DF | | T.Stat | | | p | | | p<.05 | |
| Near.Side | | Near.Top | | | | 53 | | -0.9410239 | | | 0.35096210 | | | ns | |
| Near.Side | | Far | | | | 53 | | -4.7675073 | | | 0.00001497 | | | *** | |
| Near.Top | | Far | | | | 53 | | -5.2847757 | | | 0.00000242 | | | *** | |
| 2D - Paired *t* tests  *alternative hypothesis: true mean is not equal to 0* | | | | | | | | | | | | | | | |
| Group1 | | Group2 | | | | DF | | T.Stat | | | p | | | p<.05 | |
| Near.Side | | Near.Top | | | | 53 | | -0.3794303 | | | 0.70588440 | | | ns | |
| Near.Side | | Far | | | | 53 | | -4.3732810 | | | 0.00005757 | | | *** | |
| Near.Top | | Far | | | | 53 | | -5.2621239 | | | 0.00000262 | | | *** | |

| **Distractor Attraction Difference** | | | | | | | | | | | | | | | |
| --- | --- | --- | --- | --- | --- | --- | --- | --- | --- | --- | --- | --- | --- | --- | --- |
| 3D - ANOVA | | | | | | | | | | | | | | | |
| Effect | | | DFn | DFd | | | F | | | p | | p<.05 | | | pes |
| distractor_type | | | 2 | 106 | | | 5.202 | | | 0.007 | | ** | | | 0.08900 |
| room | | | 1 | 53 | | | 0.912 | | | 0.344 | | ns | | | 0.01700 |
| distractor_type:room | | | 2 | 106 | | | 0.032 | | | 0.968 | | ns | | | 0.00061 |
| 2D - ANOVA | | | | | | | | | | | | | | | |
| Effect | | | DFn | DFd | | | F | | | p | | p<.05 | | | pes |
| distractor_type | | | 2 | 106 | | | 5.940 | | | 0.004 | | ** | | | 0.10100 |
| room | | | 1 | 53 | | | 0.545 | | | 0.464 | | ns | | | 0.01000 |
| distractor_type:room | | | 2 | 106 | | | 0.018 | | | 0.982 | | ns | | | 0.00033 |
|  | 3D - One sample *t* tests  *alternative hypothesis: true mean is not equal to 0* | | | | | | | | | | | | | | |
|  | Distractor | | DF | | T.Stat | | | | p | | | | p<.05 | | |
|  | Near.Side | | 53 | | 5.722555 | | | | 4.985593e-07 | | | | *** | | |
|  | Near.Top | | 53 | | 7.121304 | | | | 2.876992e-09 | | | | *** | | |
|  | Far | | 53 | | 5.833647 | | | | 3.326508e-07 | | | | *** | | |
|  | 2D - One sample *t* tests  *alternative hypothesis: true mean is not equal to 0* | | | | | | | | | | | | | | |
|  | Distractor | | DF | | T.Stat | | | | p | | | | p<.05 | | |
|  | Near.Side | | 53 | | 5.588962 | | | | 8.094680e-07 | | | | *** | | |
|  | Near.Top | | 53 | | 6.993762 | | | | 4.615632e-09 | | | | *** | | |
|  | Far | | 53 | | 5.862304 | | | | 2.996153e-07 | | | | *** | | |
| 3D - Paired *t* tests  *alternative hypothesis: true mean is not equal to 0* | | | | | | | | | | | | | | | |
| Group1 | | Group2 | | | | DF | | T.Stat | | | p | | | p<.05 | |
| Near.Side | | Near.Top | | | | 53 | | -2.130196 | | | 0.03781551 | | | * | |
| Near.Side | | Far | | | | 53 | | 1.013637 | | | 0.31536099 | | | ns | |
| Near.Top | | Far | | | | 53 | | 3.288258 | | | 0.00179421 | | | ** | |
| 2D - Paired *t* tests  *alternative hypothesis: true mean is not equal to 0* | | | | | | | | | | | | | | | |
| Group1 | | Group2 | | | | DF | | T.Stat | | | p | | | p<.05 | |
| Near.Side | | Near.Top | | | | 53 | | -2.3320578 | | | 0.02352952 | | | * | |
| Near.Side | | Far | | | | 53 | | 0.9358794 | | | 0.35358019 | | | ns | |
| Near.Top | | Far | | | | 53 | | 3.4812637 | | | 0.00100765 | | | ** | |

| **Cluster Based Permutation** | | | | | |
| --- | --- | --- | --- | --- | --- |
| 3D – Target Attraction Difference | | | | | |
| Distractor Type | p | Mass | Start | End | Size |
| Near.Side | 0.000 | 434 | 1 | 79 | 79 |
| Near.Top | 0.000 | 381 | 1 | 72 | 72 |
| Far | 0.018 | 50 | 32 | 50 | 19 |
| 2D – Target Attraction Difference | | | | | |
| Distractor Type | p | Mass | Start | End | Size |
| Near.Side | 0.000 | 398 | 1 | 75 | 75 |
| Near.Top | 0.000 | 374 | 1 | 72 | 72 |
| Far | 0.014 | 56 | 32 | 52 | 21 |
| 3D – Distractor Attraction Difference | | | | | |
| Distractor Type | p | Mass | Start | End | Size |
| Near.Side | 0.000 | 302 | 1 | 78 | 78 |
| Near.Top | 0.000 | 349 | 1 | 72 | 72 |
| Far | 0.000 | 234 | 1 | 53 | 53 |
| 2D – Distractor Attraction Difference | | | | | |
| Distractor Type | p | Mass | Start | End | Size |
| Near.Side | 0.000 | 295 | 1 | 79 | 79 |
| Near.Top | 0.000 | 325 | 1 | 65 | 65 |
| Far | 0.000 | 233 | 1 | 53 | 53 |
| 3D – Other Attraction Difference | | | | | |
| Distractor Type | p | Mass | Start | End | Size |
| Near.Side | 0.001 | 252 | 1 | 58 | 58 |
| Near.Top | *No significant clusters* | | | | |
| Far | 0.001 | 109 | 1 | 31 | 31 |
| 2D – Other Attraction Difference | | | | | |
| Distractor Type | p | Mass | Start | End | Size |
| Near.Side | 0.000 | 250 | 1 | 58 | 58 |
| Near.Top | *No significant clusters* | | | | |
| Far | 0.000 | 106 | 1 | 32 | 32 |
